# Neural circuits for decision-making based on pineal photoreception in zebrafish

**DOI:** 10.1073/pnas.2520290123

**Published:** 2026-03-31

**Authors:** Seiji Wada, Yuki Yamamoto, Tomoka Saito, Masahiko Hibi, Mitsumasa Koyanagi, Akihisa Terakita

**Affiliations:** ^a^Department of Biology, Graduate School of Science, Osaka Metropolitan University, Osaka 558-8585, Japan; ^b^The Advanced Research Institute for Natural Science and Technology, Osaka Metropolitan University, Osaka 558-8585, Japan; ^c^Department of Biological Science, Graduate School of Science, Nagoya University, Furo, Chikusa, Nagoya, Aichi 464-8602, Japan

**Keywords:** opsin, pineal organ, two-photon imaging

## Abstract

Nonmammalian vertebrates possess photosensitive pineal organs. We previously found that in the zebrafish pineal organ, photoreceptor cells expressing parapinopsin 1 (PP1), a bistable opsin, exhibit “chromatic” responses to ultraviolet (UV) and visible light through a unique mechanism based on the photo-interconvertibility between UV-sensitive (inactive) and visible light-sensitive (active) states of PP1. However, it remains unclear whether PP1-based chromatic responses are transmitted to pineal ganglion cells and further to the brain. In this study, we designed light stimuli by exploiting the spectral properties of the two states of PP1 and found that pineal ganglion cells exhibited chromatic responses, characterized by opposing changes to UV and visible light, using calcium imaging. These responses were significantly reduced in PP1-deficient fish compared to wild-type fish, indicating that PP1-derived signals are transmitted to pineal ganglion cells. This spectral stimulation approach enables selective analysis of PP1-specific responses. Using these stimuli, we further identified the tegmentum as a central brain region receiving pineal-derived “color” information, although it is also indirectly influenced by retinal photoreception. Both PP1-deficient fish and tegmentum neuron-ablated fish showed impaired vertical movements in response to sequential wavelength component changes. These results suggest that the neural circuit from the pineal organ to the tegmentum, which processes PP1-based chromatic information, contributes to the decision-making process for wavelength-dependent vertical behavior that is also driven by visual input. These findings provide insights into the neural circuits integrating pineal and retinal light information and how PP1-based color opponency affects behavior via tegmentum neurons.

## Significance

Although pineal light information has long been assumed to be transmitted to the brain, functional evidence has been lacking. To identify the brain region responsible for processing pineal-derived light signals, we exploited the distinct spectral properties of the inactive (ultraviolet-sensitive) and active (visible light-sensitive) states of parapinopsin 1, a pineal bistable opsin that alone generates color opponency in the zebrafish pineal organ. Using two-photon calcium imaging, we identified the tegmentum, a region also indirectly innervated by the retina, as receiving light signals mediated by parapinopsin 1. Furthermore, we found that a neural circuit integrating inputs from the pineal organ and the eyes mediates upward and downward movements in zebrafish larvae in response to changes in light wavelength via tegmentum neurons.

Vertebrates, from cyclostomes to birds, possess photosensitive pineal-related organs. One of the physiological functions of pineal photoreception is the regulation of melatonin synthesis and secretion in a light-dependent manner, reflecting its role as an endocrine organ (1–3). On the other hand, many pineal-related organs from cyclostomes to reptiles contain neurons (pineal ganglion cells) that transmit light information to the brain in a manner similar to retinal ganglion cells (4). The photoresponses in pineal ganglion cells are classified into two types: “achromatic” and “chromatic” responses (5–8). Achromatic responses are defined as inhibition of neural firing with maximum sensitivity in the visible light region, while chromatic responses are antagonistic responses, exhibiting inhibition and promotion of neural firing in response to shorter- and longer-wavelength light, respectively. We previously found that, in the zebrafish pineal organ, parapinopsin 1 (PP1), a bistable opsin, generates color opponency through its photoreception alone at the single photoreceptor cell level (9). A photoequilibrium between two stable states, the UV-sensitive dark state (inactive state) and the visible light-sensitive photoproduct (active state activating a G protein), shifts depending on the spectral distribution of environmental light. This shift enables a single kind of opsin-based “chromatic” responses. Therefore, we speculated that PP1-evoked chromatic responses are transmitted to ganglion cells. Previous histological reports suggested that pineal ganglion cells transmit light information from the photoreceptor cells to the brain using neural tracers (4). However, there is no direct evidence demonstrating that pineal color information is functionally or physiologically transmitted to the brain via pineal ganglion cells. Furthermore, it remains unclear whether the characteristics of PP1-evoked chromatic responses observed in pineal photoreceptor cells are preserved in pineal ganglion cells and the brain.

To address this, we conducted whole-brain calcium imaging in zebrafish that pan-neuronally express GCaMP in order to visualize brain regions receiving light-driven information from the pineal organ by applying light stimuli that target the organ. However, even with laser scanning limited to the pineal organ, photoresponses from the eyes were also elicited, as the tectum and habenula, both of which receive retinal light information (10–16), reproducibly showed changes in calcium levels. This is presumably caused by diffused light during laser scanning and the high sensitivity of the eyes to light. To overcome this, we established a light-irradiation protocol based on the bistable nature of PP1 to visualize brain regions receiving pineal light information in intact fish. In addition to evaluating the transmission of chromatic responses from PP1 cells to pineal ganglion cells, we sought to verify whether light information captured in the pineal organ is transmitted to the brain, and how this information affects light-dependent behaviors.

## Results

### Light Irradiation Protocol Generating “Pineal-Specific” Events Based on PP1 Molecular Properties.

First, we attempted whole-brain calcium imaging using *Tg(elavl3(Huc):GCaMP6s)* zebrafish, where GCaMP is expressed pan-neuronally (17, 18). The region of interest (ROI) for light stimuli targeting the pineal area was scanned using a 588-nm laser from the dorsal side of the fish. The results showed strong reproducibility of photoresponses in a large number of neurons, including those in the tectum and habenula. These regions are well known to receive light information from the eyes (10–16), suggesting that diffused light stimulated the eyes. Most of the brain regions visualized were plausibly considered to be retina-associated circuits (*SI* *Appendix*, Fig. S1 and Movie S1). Therefore, to obtain evidence that pineal-derived light information is transmitted to the brain, the visualization of pineal-specific events was considered as a valid approach. We focused on the molecular property of a pineal-specific bistable opsin, PP1. The inactive state (dark state) of PP1 is UV-sensitive. UV irradiation converts the dark state to the visible light-sensitive active state (photoproduct), which, in turn, activates cone-type transducin, Gt2 (19). The photoproduct reverts to the original dark state upon absorption of visible light (9, 20, 21). These two stable states, the dark state and active photoproduct form a photoequilibrium in response to light wavelength.

**Fig. 1.**
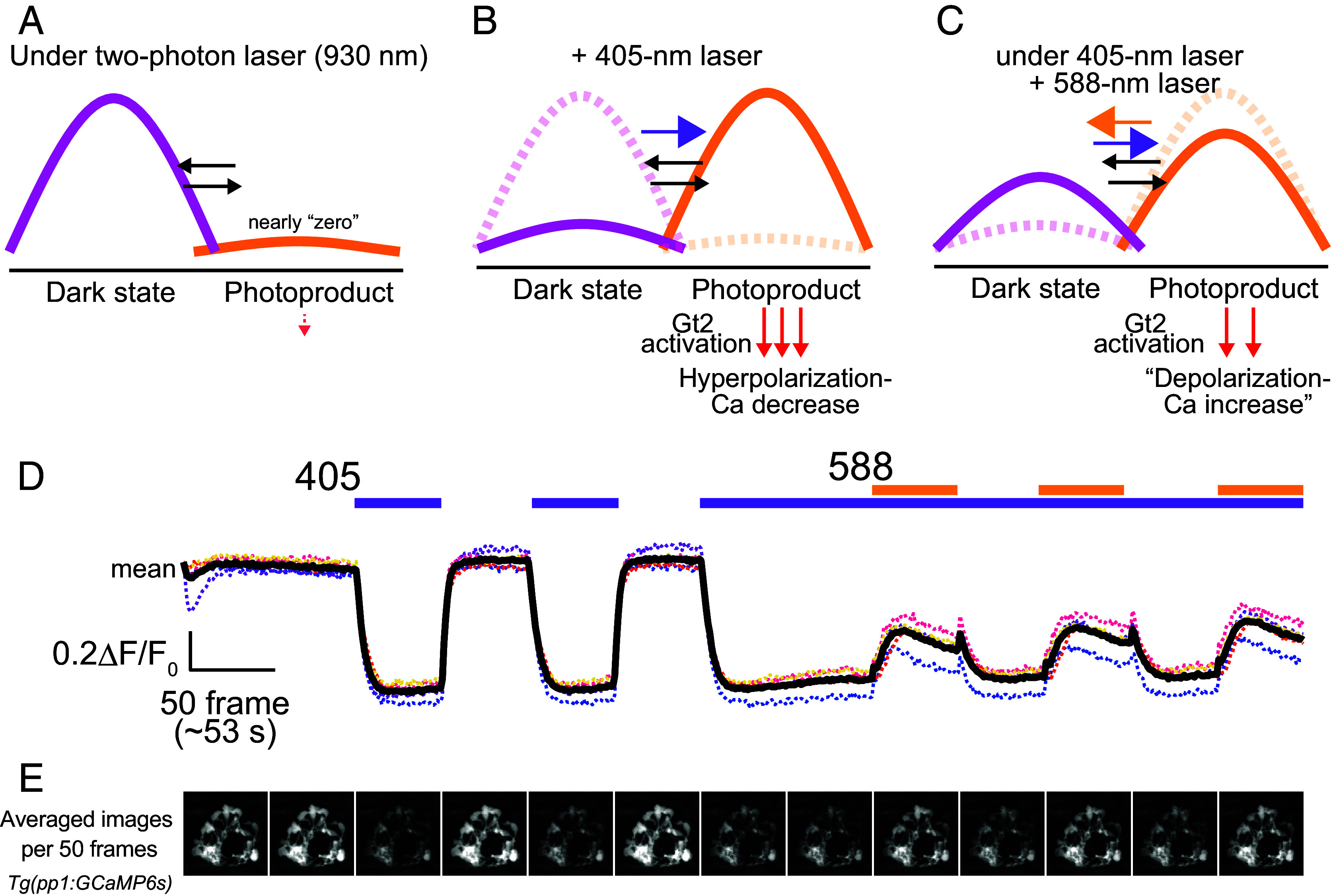
Light irradiation protocol generating PP1-specific responses in wild-type larvae. (*A*–*C*) Schematic representations illustrating the photoequilibrium between the two states of bistable PP1. (*D*) Calcium change profiles in PP1-expressing cells of *Tg(pp1:GCaMP6s)* larvae (5 to 6 dpf) in response to 405-nm (UV, purple bars) and 588-nm (visible light, yellow bars) laser stimuli, recorded using two-photon imaging. Bold and dotted traces indicate the mean and individual profiles, respectively (*n* = 5). Fluorescence intensities were normalized to the initial values. Spike-like calcium increases following visible-light exposures are due to apparatus lag time (transient darkness) during light-condition switching. (*E*) Averaged images (dorsal view) showing GCaMP fluorescence in PP1-expressing cells of the pineal organ in *Tg(pp1:GCaMP6s)* larvae over 50 frames. The images represent areas of 80 × 80 μm.

The first aim of this study was to identify projection targets of PP1 cells, including pineal ganglion cells and downstream brain regions, using pineal-specific responses based on the molecular properties of PP1 as indicators. Accordingly, we used XYZT imaging in this study, which is well suited for exploratory scanning of the pineal organ and the entire brain. Unlike the local single-plane imaging used in our previous study (9), this approach minimizes sustained PP1 activation by two-photon excitation light (Fig. 1*A*) and maximizes the dynamic range of PP1 cell responses to UV light. UV light generates visible light-sensitive active photoproducts of PP1 (Fig. 1*B*), leading to hyperpolarization. Under continuous UV irradiation, Gt2 activation induced by the photoproduct is expected to be inhibited by additional visible light irradiation, resulting in the “cancellation” of hyperpolarization, that is, “depolarization” (Fig. 1*C*). In other words, the photoequilibrium between the two states of PP1 established under UV alone (Fig. 1*B*) is predicted to shift to a new equilibrium under combined UV and visible light illumination (Fig. 1*C*).

We next examined whether this event occurs through pineal imaging using *Tg(pp1:GCaMP6s)* zebrafish (9). UV stimuli with a 405-nm laser induced a substantial decrease in calcium levels, accompanied by hyperpolarization (Fig. 1 *D* and *E* and Movie S2). This UV-induced Ca^2+^-decrease was robustly and reproducibly observed upon repeated UV stimuli. Additional visible light stimuli using a 588-nm laser were applied while maintaining UV stimulation, which reproducibly elicited calcium increases. We then confirmed whether this calcium change was caused by a chromatic reaction arising from the molecular property of PP1 (*SI* *Appendix*, Fig. S2). Specifically, we examined whether the presence or absence of background UV differently alters the response to visible light in PP1^+/−^ and PP1^−/−^ fish. In PP1^+/−^ fish, visible-light exposure in the absence of UV elicited a slight decrease in calcium level rather than an increase, whereas in the presence of UV, it elicited a robust calcium increase comparable to that of wild-type fish (*SI* *Appendix*, Fig. S2, PP1^+/−^; Fig. 1*D*). This UV-dependent alteration of the response to visible light can be explained by shifts in the relative proportions of the two PP1 states, that is, by shifts in the PP1 photoequilibrium (Fig. 1 *A*–*C*). The mechanism underlying the visible light-induced calcium decrease in PP1 cells under UV-free conditions remains unclear (see *SI* *Appendix*, Fig. S2 legend). In contrast, only residual color opponent-like responses were observed in PP1^−/−^ fish, characterized by a small, gradual decrease under UV and a slight increase under visible light, which may be mediated by unknown opsins. Since the difference in responses to visible light with or without background UV in PP1^−/−^ fish was substantially small compared to that in PP1^+/−^ fish, the response to visible light observed under UV background conditions in PP1^+/−^ fish indicates a chromatic response based on the molecular properties of PP1. Thus, we used this light irradiation protocol as a valid and effective method to elicit PP1-photoreception-specific events.

### A Subset of Pineal Ganglion Cells Exhibits a “Color” Response Evoked by PP1.

**Fig. 2.**
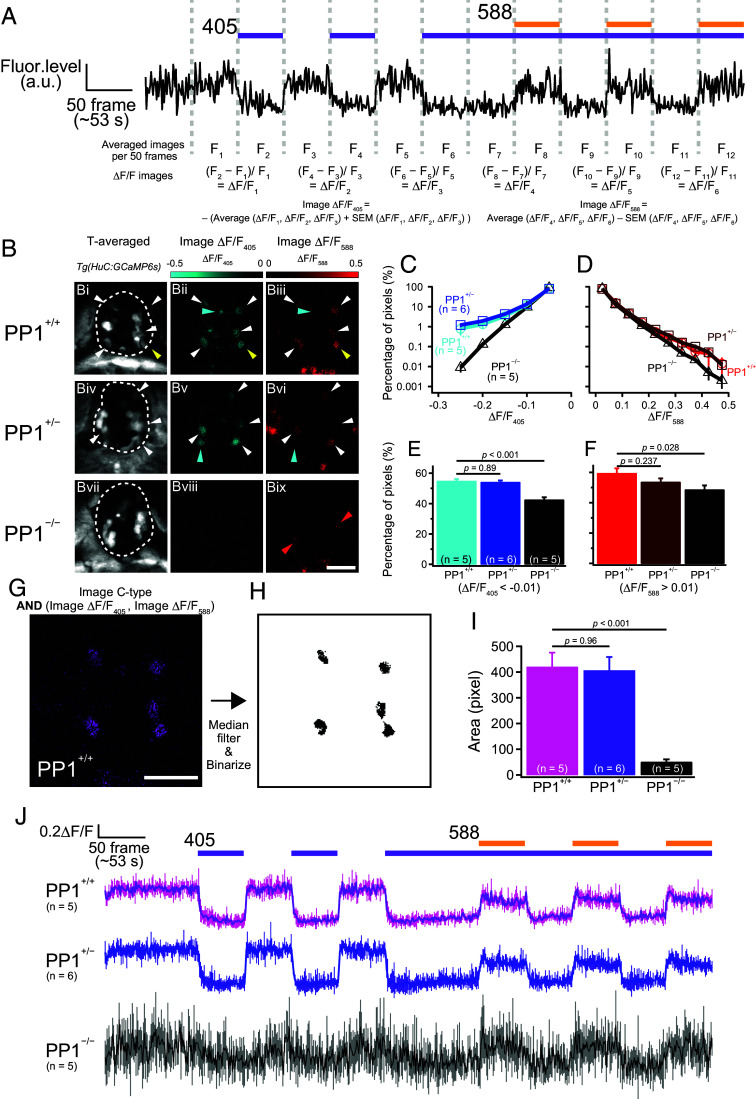
Pineal ganglion cells showing chromatic properties originating from PP1 cells. (*A*) Calcium change profile in a single pineal ganglion cell exhibiting a pattern similar to that of PP1 cells in *Tg(elavl3:GCaMP6s)* larvae. (*B*) Image analyses showing the calcium level decrease by UV (image ΔF/F_405_) and the calcium level increase by visible light (image ΔF/F_588_) across different genotypes: PP1^+/+^ (*Bi*–*Biii*), PP1^+/−^ (*Biv*–*Bvi*), and PP1^−/−^ (*Bvii*–*Bix*). T-averaged images represent the average of all frames. Yellow arrowheads represent the pineal ganglion cell showing the calcium change profile in (*A*). White arrowheads indicate cells showing a decrease by UV and an increase by visible light. Cyan arrowheads indicate cells showing only a UV-induced decrease. Red arrowheads indicate cells showing a small visible light-induced calcium increase. The dotted landmarks indicate the ROIs used for the pixel-based analyses in (*C*–*F*). (*C* and *D*) Comparison of pixel-based histograms for ΔF/F_405_ and ΔF/F_588_ across different genotypes, PP1^+/+^ [*n* = 5, blue (*C*) and red (*D*) circles], PP1^+/−^ [*n* = 6, light blue (*C*) and brown (*D*) squares], and PP1^−/−^ (*n* = 5, black triangles). The vertical axis indicates the percentage of pixels within the pineal ROI shown in (*B*) (e.g., *Bi*, *Biv*, and *Bvii*) that fall into each ΔF/F range. (*E* and *F*) Quantification of pixel values in images ΔF/F_405_ and ΔF/F_588_ (Dunnett’s multiple comparison test). (*G*, *H*, *I*, and *J*) Visualization of chromatic-type (C-type) ganglion cells (*G*), followed by a binary image (*H*) used for quantitative analyses of the C-type area (*I*, Dunnett’s multiple comparison test) and the ROI for obtaining the C-type calcium change profile (*J*). (Scale bar: 25 μm.) Experiments were performed using 5 to 6 dpf zebrafish larvae.

Next, we tested if pineal ganglion cells exhibit an antagonistic response to UV and visible light stimuli based on the bistable properties of PP1. Using *Tg(elavl3(HuC):GCaMP6s)* zebrafish, where GCaMP is expressed in pineal ganglion cells but not in photoreceptor cells (22), we performed pineal calcium imaging using the established light irradiation protocol. We found that calcium levels in several ganglion cells decreased in response to UV light and increased in response to visible light (Fig. 2*A*). These antagonistic properties in ganglion cells are similar to those in PP1 cells (Fig. 1*D*). To evaluate whether this antagonistic response results from ganglion cells receiving light information from PP1 cells, we conducted comparative image analyses using homozygous knockout (PP1^−/−^), heterozygous knockout (PP1^+/−^), and wild-type (PP1^+/+^) zebrafish (Fig. 2*B* and *SI* *Appendix*, Fig. S3). From the averaged images of 50 frames (shown as F_1_ to F_12_), ΔF/F images were generated. Three repetitions of each UV (405-nm) and visible light (588-nm) stimulus allowed us to create images showing the average and SE for each of the three ΔF/F images. The ΔF/F_405_ and ΔF/F_588_ images were constructed by subtracting the two images showing the average and SE to avoid overestimating nonspecific calcium changes. In the images from PP1^+/+^ and PP^+/−^ fish, ganglion cells that responded to UV and visible light stimuli were clearly visualized (Fig. 2 *B**, i*–*vi*). In contrast, images from PP1^−/−^ fish did not show obviously visualized cells (Fig. 2 *B*, *vii*–*ix*). We analyzed the pixel-based histograms of ΔF/F values in the pineal area from the ΔF/F_405_ and ΔF/F_588_ images. The histograms for PP1^+/+^ and PP^+/−^ fish were similar, while those for PP1^−/−^ fish differed significantly from the others (Fig. 2 *C* and *D*). In addition, the number of pixels exhibiting ΔF/F values above a 1% change rate was significantly lower in PP1^−/−^ fish compared to PP1^+/+^ and PP1^+/−^ fish (Fig. 2 *E* and *F*). These results suggest that large calcium changes in response to UV and visible light stimuli in pineal ganglion cells require PP1 photoreception. Moreover, most of the visualized cells exhibited a calcium response to both stimuli (Fig. 2 *B*, *i*–*vi*, white arrowheads). To confirm that the antagonistic response in these cells was indeed derived from PP1 cells, we created images showing “chromatic”-type (C-type) ganglion cells, which reproducibly exhibit decreases and increases in calcium levels in response to UV and visible light, respectively (Fig. 2*G*). After further image processing (Fig. 2*H*), we measured the area. In PP1^−/−^ fish, the C-type area was significantly reduced compared with PP1^+/+^ fish (Dunnett’s multiple comparison test, *P* < 0.001; Fig. 2*I*). The calcium change profiles from the ROIs generated by image processing in Fig. 2*H* showed an evident antagonistic response in the pineal ganglion cells of PP1^+/+^ and PP1^+/−^ fish, with similar patterns between the two genotypes. In contrast, the response in PP1^−/−^ fish was ambiguous and markedly different from those in the other two groups, although a small number of residual responses showing color opponency were still observed in PP1^−/−^ fish (Fig. 2 *I* and *J*). These observations indicate that some pineal ganglion cells receive light information from PP1 cells.

### Pineal Light Information Is Transmitted to the Tegmentum in the Midbrain.

**Fig. 3.**
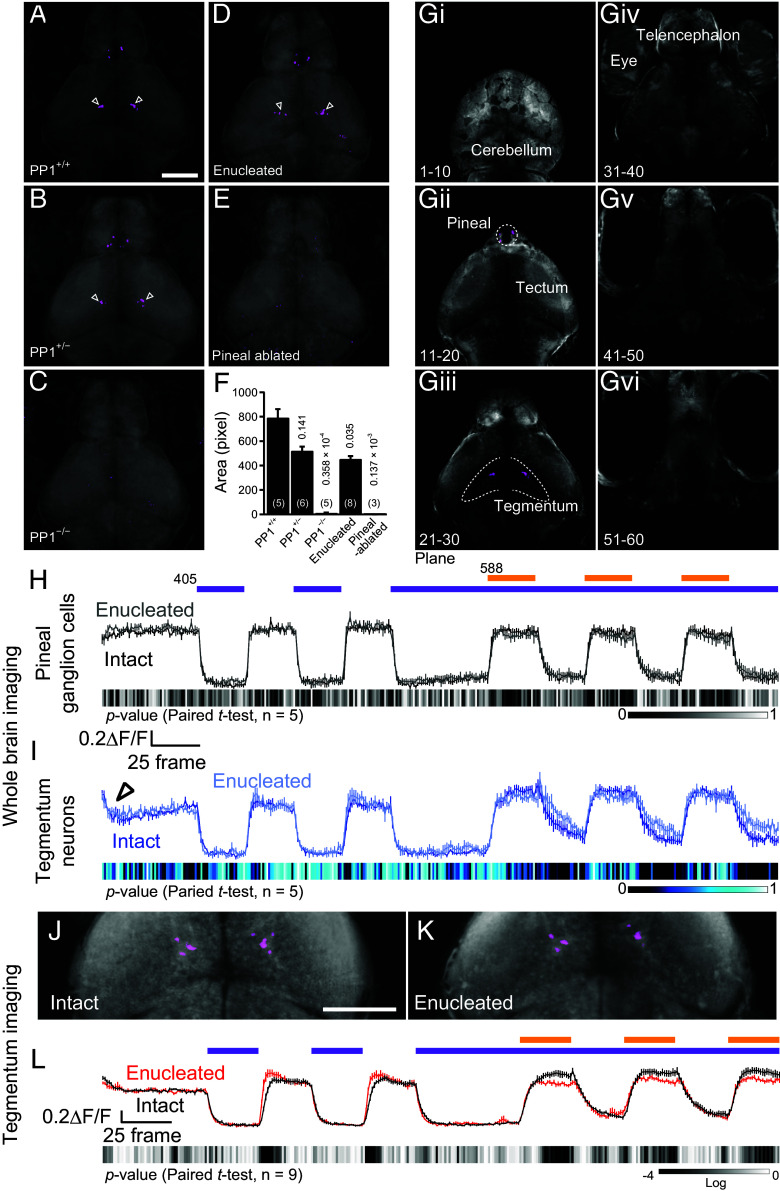
The pineal C-type property based on PP1 is transmitted to tegmentum neurons. (*A*–*E*) C-type neuron-visualized images in different genotypes: PP1^+/+^ (*A*), PP1^+/−^ (*B*), and PP1^−/−^ (*C*), eye-enucleated fish (*D*), and pineal-ablated fish (*E*) in whole-brain imaging. (Scale bar: 100 μm.) (*F*) Quantification of the C-type area in the tegmentum (Dunnett’s multiple comparison test). (*Gi*–*Gvi*) Six separate optical brain sections showing that C-type pineal ganglion cells (*Gii*) and C-type tegmentum neurons (*Giii*) are present in different planes and that C-type cells were not found in other planes (*Gi* and *Giv*–*vi*). (*H* and *I*) Comparison of calcium change profiles in C-type pineal ganglion cells (*H*) and tegmentum neurons (*I*) before (intact, black and blue) and after (enucleated, gray and light blue) surgical enucleation (*n* = 5) in whole-brain imaging. The open arrowhead in (*I*) shows the decrease in calcium levels upon the onset of two-photon excitation laser in the tegmentum neurons. (*J*, *K*, and *L*) Comparison of C-type-visualized images (*J* and *K*) and calcium change profiles (*L*) before and after surgical enucleation (*n* = 9) in tegmentum imaging. Data are normalized to the average of frames 26-50 (25 frames before the initial UV exposure in *H*, *I*, and *L*). (Scale bar: 100 μm.) Experiments were performed using 5 to 6 dpf zebrafish larvae.

We next investigated brain regions that receive pineal light information using the established light irradiation protocol in whole-brain imaging. The imaging procedure was performed with three steps of XYZT imaging (i.e., planes 1 to 20, 21 to 40, and 41 to 60) to avoid differences in extreme time resolution between pineal and whole-brain imaging (Fig. 3 *G*, *i*–*vi*). Using the same image processing method to visualize C-type pineal ganglion cells, we examined the location of neurons exhibiting the “chromatic” property. As a result, we identified C-type neurons in the tegmentum, a region in the midbrain, as well as in pineal ganglion cells in PP1^+/+^ and PP1^+/−^ fish (Fig. 3 *A* and *B*). In contrast, PP1^−/−^ fish showed no visualized C-type neurons in the tegmentum (Fig. 3*C*). To determine whether the majority of the C-type responses in the tegmentum are derived from pineal PP1 photoreception, we performed imaging on surgically eye-enucleated fish and pineal-ablated fish. We found that C-type tegmentum neurons were clearly visualized in enucleated fish but not in pineal-ablated fish (Fig. 3 *D* and *E*). These observations demonstrate that the majority of PP1-based chromatic information is transmitted to a subset of tegmentum neurons. However, retinal photoreception may also play a role, as indicated by the decreased C-type area in enucleated fish compared to intact (PP1^+/+^) fish (Fig. 3*F*). The tegmentum C-type neurons exhibited light-dependent calcium change profiles that were similar to, but not identical with, those of the C-type pineal ganglion cells (Fig. 3*H*, black trace and Fig. 3*I*, blue trace). Calcium decreases were observed at the initial step under two-photon laser scanning in the tegmentum neurons (Fig. 3*I*, open arrowhead), but not in the pineal ganglion cells. After visible light exposure, the calcium changes in the pineal ganglion cells showed more rapid decreases compared to those in the tegmentum neurons. This difference suggests that additional neural circuits other than those involving PP1-based color opponency may be present.

### C-Type Tegmentum Neurons Are Affected by Neural Circuits Associated with Retinal Photoreception.

To test the possibility that neural circuits involving retinal photoreception are involved in the output of C-type tegmentum neurons, we compared the calcium change profiles of these neurons between intact and enucleated fish. The calcium change profiles in pineal ganglion cells were almost identical between the two groups (Fig. 3*H*). However, after the termination of visible light exposure, the tegmentum neurons exhibited slower calcium decay kinetics in enucleated fish compared to intact fish (Fig. 3*I*). As explained above, whole-brain imaging was carried out in three separate layers at different “z”-positions (planes 1 to 20, 21 to 40, and 41 to 60, 4.5 μm per step, totaling 270 μm depth). In our imaging procedure, two-photon laser scanning was simultaneously performed to acquire images of both the C-type tegmentum neurons in planes 21 to 30 and the eyes in planes 31 to 40. Activation of opsins in the eyes and brain by background two-photon laser scanning may have prevented intact fish from fully conveying retinal light responses to C-type tegmentum neurons under UV and visible light stimulation. To minimize excessive opsin activation caused by two-photon excitation, we performed imaging restricted solely to the tegmentum. The imaging area was set to cover the tegmentum, while the light stimuli were applied to the top of the pineal organ, outside the imaging area. When comparing C-type neuron images before and after enucleation, no remarkable differences were observed (Fig. 3 *J* and *K*). However, during visible-light exposure, the increase in calcium level was greater in intact fish than that in enucleated fish (Fig. 3*L*). The slight difference in the kinetics of the calcium increase immediately after UV light offset observed in whole-brain imaging (Fig. 3*I*) became clearer in tegmentum imaging (Fig. 3*L*). These observations suggest that C-type tegmentum neurons integrate light information from both pineal PP1 and retinal opsins. Additionally, in enucleated fish, calcium decay following the offset of visible light was relatively rapid in tegmentum imaging compared with whole-brain imaging, suggesting that the responses of C-type neurons may be modified by light information derived from nonvisual opsins expressed in the brain (23).

### Symmetric Ipsilateral Innervation of C-Type Tegmentum Neurons from the Pineal Organ.

**Fig. 4.**
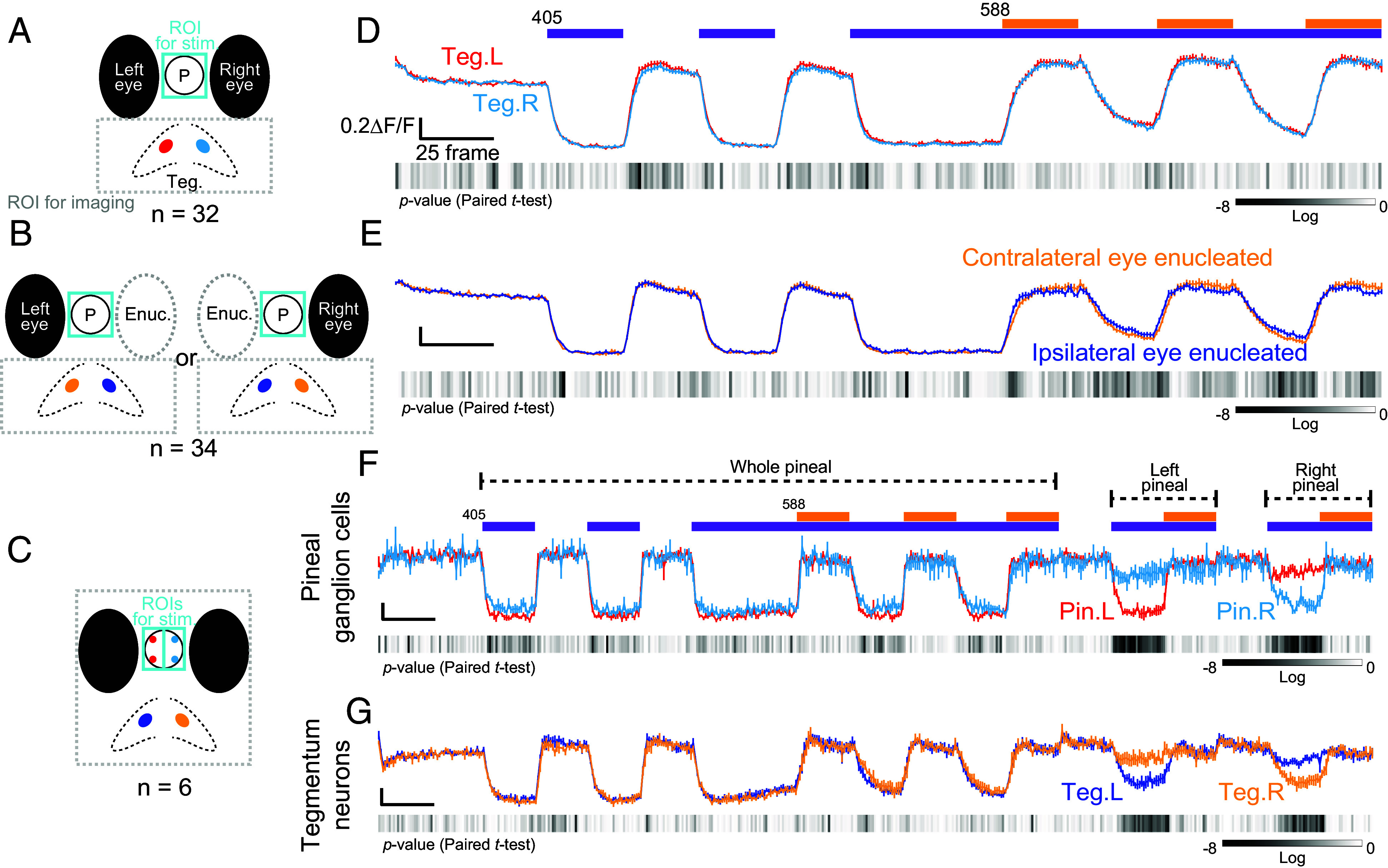
Relationship between photosensory organs and tegmentum in innervation for C-type neurons. (*A*–*C*) Schematic representations of the targeted neurons to examine how they are innervated by the eyes and the pineal organ. P, pineal organ; Teg, tegmentum; Enuc, enucleated eye. The C-type neurons in the colored areas were comparatively analyzed in (*D*–*G*). (*D* and *E*) Calcium change profiles recorded in tegmentum imaging. Colored profiles represent data obtained from left (red) and right (cyan) C-type tegmentum neurons (*D*) and C-type tegmentum neurons in contralateral (yellow) and ipsilateral (blue) eye-enucleated fish (*E*). (*F* and *G*) Calcium change profiles recorded in whole-brain imaging from limited planes (refer to 11-30 planes in Fig. 3 *G**, ii* and *iii*), with additional stimuli applied to the left and right pineal organ. The profiles show calcium changes in the left (red) and right (cyan) pineal ganglion cells (*F*), and in the left (blue) and right (yellow) tegmentum neurons (*G*). Experiments were performed using 5 to 6 dpf zebrafish larvae.

In zebrafish, the optic nerves from the left and right eyes cross on the ventral side of the brain and contralaterally innervate the optic tectum and pretectum (24, 25). Here, we examined if the same contralateral innervation applies to the relationship between the eyes and C-type tegmentum neurons. The calcium change profiles in C-type tegmentum neurons on the left and right sides are not largely different from each other, suggesting that these neurons are symmetrically innervated by both the pineal organ and the eyes (Fig. 4 *A* and *D*). To further investigate how the C-type tegmentum neurons are innervated by the lateral eyes, we used one eye-enucleated fish. The calcium change profiles in the C-type tegmentum neurons between ipsilateral and contralateral eye-enucleated fish were nearly identical, indicating that retinal innervation to C-type tegmentum neurons is not strongly biased toward either the ipsilateral or contralateral side (Fig. 4 *B* and *E*). These findings suggest that spatial information obtained from the left and right eyes does not largely affect the C-type tegmentum neurons.

Next, we aimed to determine the significance of the separation and transmission of PP1-based responses to the left and right sides of the C-type tegmentum neurons. The pineal organ and eyes in vertebrates are considered to share an evolutionary origin because many molecules involved in photosensitivity, such as opsins, transducin, phosphodiesterase, opsin kinases, and arrestins, are shared (26–30). It is of interest if the contralateral projection pattern is also conserved. To test this, we applied the same light irradiation protocol, followed by separate light irradiation to the left and right sides of the pineal organ (Fig. 4*C* and *SI* *Appendix*, Fig. S4). C-type pineal ganglion cells and C-type tegmentum neurons were visualized using this light irradiation protocol applied to the whole pineal organ. The C-type pineal ganglion cells on the left and right sides showed strong responses to light irradiation on the corresponding side (Fig. 4*F*). In the same manner, the left and right C-type tegmentum neurons showed large responses to light irradiation on their respective sides (Fig. 4*G*). These results indicate that the innervation from PP1 cells in the pineal organ to most C-type tegmentum neurons is strongly biased ipsilaterally, suggesting that the pineal organ has spatial resolution for light.

### PP1 Photoreception Contributes to Short-Wavelength Light-Dependent Movement toward Water Depth under Blue Background Light.

**Fig. 5.**
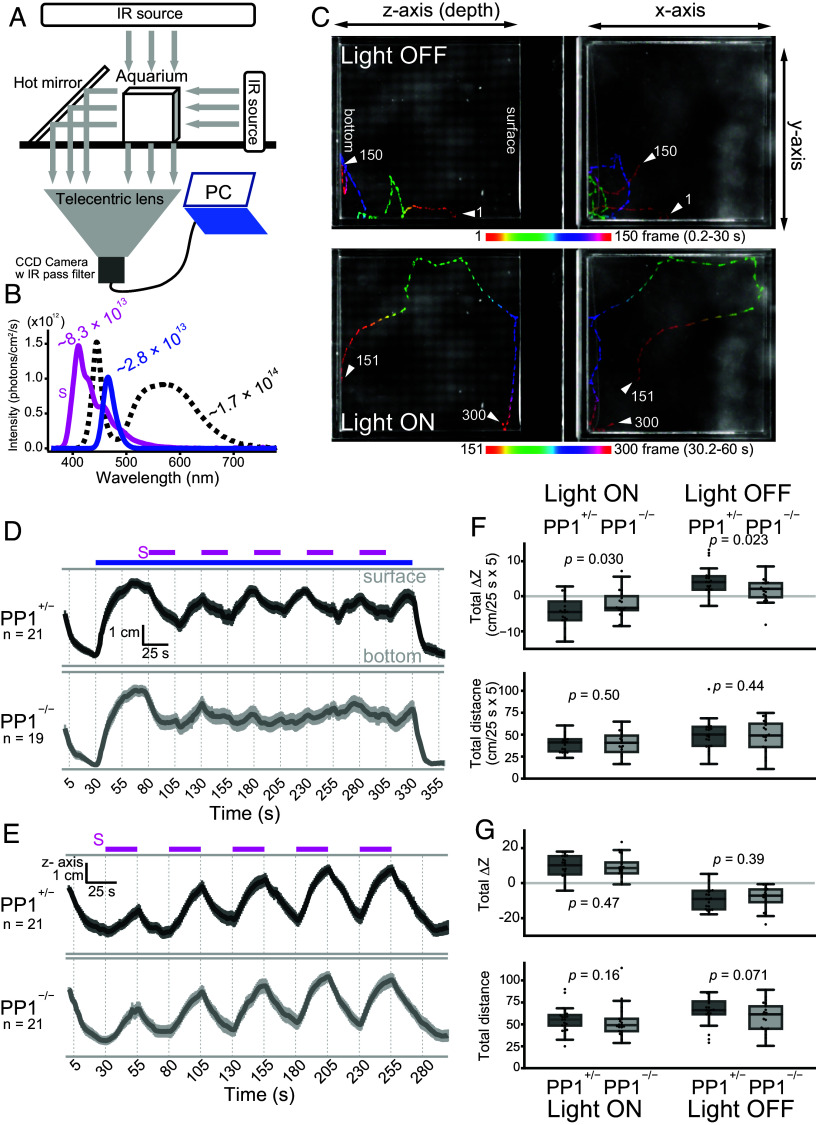
Short-wavelength light-dependent behaviors in PP1-deficient fish and siblings. (*A*) Schematic representation of the three-dimensional behavior recording system. (*B*) Spectral distributions of light used in behavior tests. Short-wavelength light (S, magenta) was used as the stimulus, while the standard white LED (dotted curve) was used to keep fish near the surface before recordings. Blue light served as the background light. (*C*) Fish trajectories during behavior involving retinal photoreception when the light turned off (*Top*) and on (*Bottom*). (*D* and *E*) Comparison of behavioral traces showing fish z-position changes during short-wavelength light exposure under conditions with (*D*) or without (*E*, i.e., dark conditions) blue background light. (*F* and *G*) Quantification and comparison of the total ΔZ (*Top*) and distance (*Bottom*) in (*D* and *E*) during onset or offset of the short-wavelength light between PP1^+/−^ and PP1^−/−^ fish (Wilcoxon rank sum test). Experiments were performed using 10 to 11 dpf zebrafish larvae.

The physiological function of the neural light information transmitted from the pineal organ to the brain remains unclear. We have previously reported that PP1-based color opponency is achieved under background light that forms an equilibrium between the UV-sensitive inactive and visible light-sensitive active states of PP1 (9). As described above, the PP1 photoequilibrium-based color opponency signals generated in PP1 cells are transmitted to the tegmentum via pineal ganglion cells. We investigated zebrafish behaviors dependent on the PP1 photoequilibrium-based color opponency using a three-dimensional recording system (Fig. 5*A*) and identified multiple reproducible behavioral patterns under different light conditions in 10- to 11-dpf wild-type larvae (*SI* *Appendix*, Fig. S5). Specifically, we focused on the behavior of zebrafish larvae in response to the onset and offset of short-wavelength light under blue background light conditions, in which the onset of short-wavelength light increases the PP1 active state, thereby shifting the PP1 photoequilibrium toward the active-state-abundant condition, whereas its offset decreases the active state, facilitating a return of the equilibrium to the active-state-scarce condition.

We comparatively analyzed the behavior of PP1^+/−^ and PP1^−/−^ zebrafish larvae (10 to 11 dpf) derived from crosses between PP1^+/−^ and PP1^−/−^ fish, under the conditions with or without blue background light. Initially, the fish were kept under white LED light (Fig. 5*B*, black dotted curve), under which most fish swam near the surface. Recordings began when the white LED light was turned off, which caused the fish to move downward (Fig. 5 *C*, *Top*), consistent with the previous report (31). After 30 s of continuous darkness, exposure to blue background light (Fig. 5*B*, blue curve) induced upward movement in PP1^+/−^ fish (Fig. 5 *C*, *Bottom*; Fig. 5*D*). When additional short-wavelength light was applied (Fig. 5*B*, magenta curve) under blue background light, the fish exhibited downward movement (Fig. 5 *D*, *Top*). In response to five cycles of short-wavelength light onset and offset under background light, PP1^+/−^ fish exhibited clear and regular downward and upward movements. In contrast, in the absence of blue background light conditions (i.e., dark conditions), the larvae exhibited different behavior in response to short-wavelength light: Onset of short-wavelength light facilitated upward movements, and its offset caused downward movements (Fig. 5 *E*, *Top*). Wild-type fish also exhibited nearly identical behaviors to those of PP1^+/−^ fish in response to the onset and offset of the short-wavelength light, both in the presence and absence of blue background light (*SI* *Appendix*, Fig. S5 *A* and *B*). Notably, in PP1^−/−^ fish, such regular movements were observed in the absence of background light and were nearly identical to those in PP1^+/−^ fish (Fig. 5 *E*, *Bottom*), but they were largely absent in the presence of blue background light (Fig. 5 *D*, *Bottom*); in other words, the short-wavelength light-dependent movements mediated by PP1 photoreception were observed only in the presence of blue background light. A quantitative difference was found in the total ΔZ values, representing changes in fish z-positions, between PP1^+/−^ and PP1^−/−^ fish; however, the total distance traveled was not significantly different (Fig. 5*F*). These results indicate that PP1 photoreception is involved in fish movement along the water-depth axis, rather than simply influencing overall activity levels. Importantly, the behavioral phenotype involving PP1 strongly depends on background light that forms a photoequilibrium between the inactive and active states of PP1 (Fig. 5 *D*–*G*). Thus, our findings suggest that decision-making about whether to swim up or down in zebrafish larvae is mediated by information derived from an equilibrium shift between the two PP1 states. This study demonstrates an example of PP1 photoreception triggering specific behaviors.

We further investigated whether the onset and offset of long-wavelength irradiation, which decreases the active state and facilitates a shift of the PP1 photoequilibrium from the active state-scarce conditions to the active-state-nearly-zero conditions, under blue background light, elicits opponent behaviors mediated by a PP1 photoequilibrium shift. However, no clear differences were detected between PP1^+/−^ and PP1^−/−^ fish (*SI* *Appendix*, Fig. S6; see the *Discussion* section).

### Vertical Movement Is Mediated by the Neural Pathway from Pineal PP1 Cells to C-Type Neurons in the Tegmentum.

**Fig. 6.**
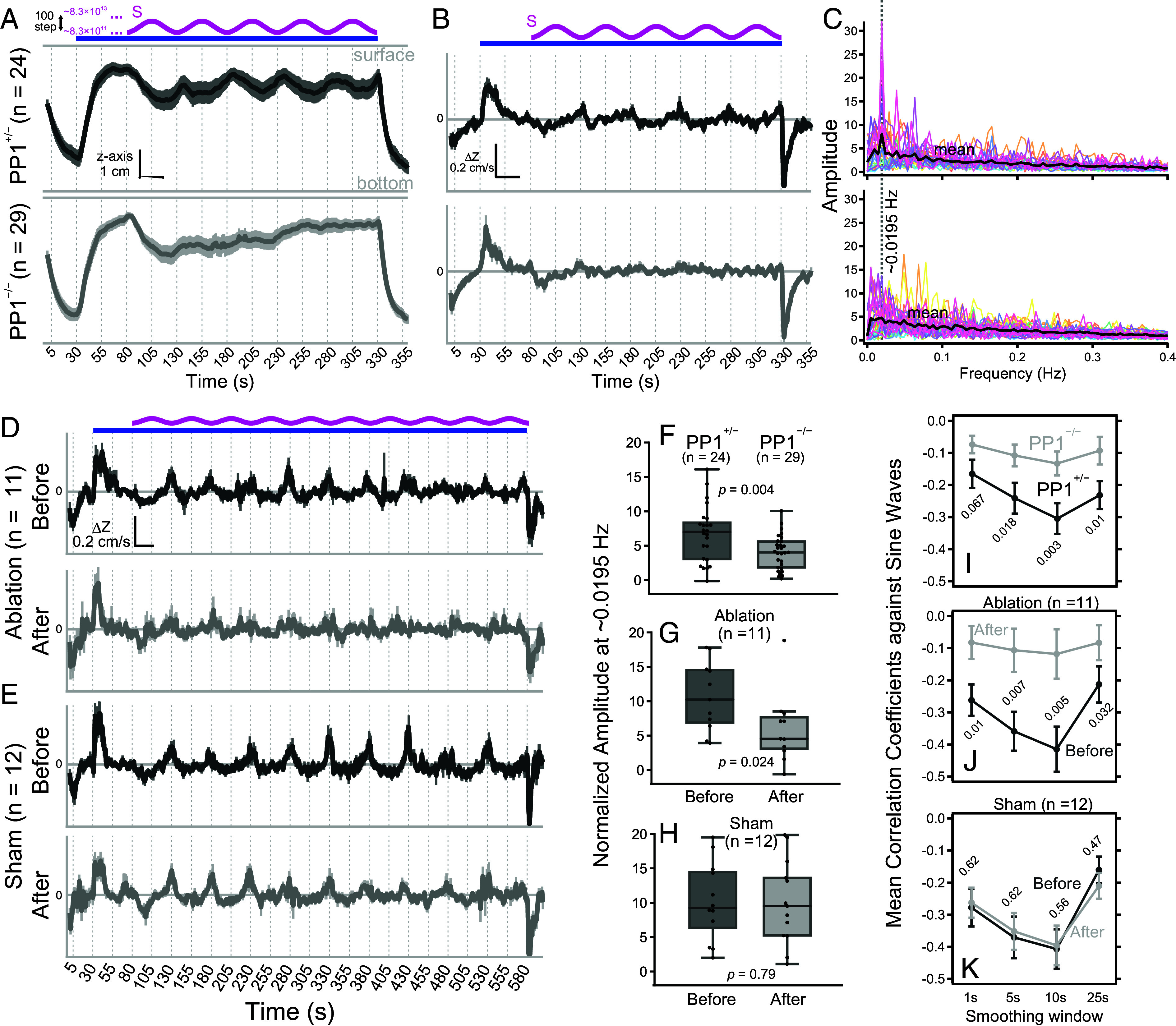
PP1-related behavior changes against gradual intensity changes following sine waves. (*A* and *B*) Comparison of behavioral traces showing fish z-position changes and the amount of behavior change along the z-axis per second (ΔZ) in response to sine wave-followed short-wavelength light changes under a blue background, between PP1^+/−^ (*Top*) and PP1^−/−^ (*Bottom*) fish. (*C*) Power spectra showing periodicity in ΔZ through FFT analyses. (*D* and *E*) Comparative analyses of ΔZ before (*Top*) and after (*Bottom*) laser ablation of C-type tegmentum neurons (*D*) or sham operation (*E*). (*F*–*K*) Quantification of periodicities in ΔZ (*F*–*H*) and the correlation coefficients between sine wave-followed short-wavelength light intensity and smoothed-ΔZ (*I*–*K*). Comparison of these between PP1^+/−^ and PP1^−/−^ fish (*F* and *I*, Wilcoxon rank sum test), before and after laser ablation of C-type tegmentum neurons (*G* and *J*, paired *t* test), and after sham operation (*H* and *K*, paired *t* test). Experiments were performed using 10 to 11 dpf zebrafish larvae.

To obtain further evidence that PP1-dependent behaviors involve color opponency based on the PP1 photoequilibrium, we set up an experiment to examine whether these behaviors are attenuated by ablation of C-type neurons in the tegmentum, to which PP1 photoequilibrium-based color-opponent signals project (Fig. 3). However, PP1^−/−^ fish exhibited small residual behavioral changes in response to repeated light stimuli as well as to the initial onset of light under blue background conditions (Fig. 5 *D*, *Bottom*), which suggests that photoreception by opsins other than PP1 also contributes to the vertical movements. Residual responses observed in pineal ganglion cells of PP1^−/−^ fish (Fig. 2*J*), likely mediated by opsins other than PP1, might be one of the candidates explaining the remaining behaviors observed under blue background light in PP1^−/−^ larvae. To accurately assess the effects of C-type neuron ablation in the tegmentum, it is necessary to “isolate” and analyze behaviors specifically attributable to color opponency based on shifts in PP1 photoequilibrium. Considering the possibility that this residual behavior under blue background light in PP1^−/−^ fish could be induced by an unknown opsin through rapid changes in light intensity, we attempted to isolate upward and downward movements dependent solely on color opponent signals based on shifts in PP1 photopigment equilibrium by gradually varying the intensity of additional short-wavelength irradiation. Specifically, by using gradual light-intensity changes following 0.02 Hz sine waves, we found that upward and downward behaviors based on PP1 photoreception were elicited. The mean traces for PP1^+/−^ fish show z-position changes that closely follow the sine wave (Fig. 6 *A*, *Top*). In contrast, PP1^−/−^ fish did not exhibit clear behavior patterns in response to sine wave light changes, although they did show downward movement during the initial wave (Fig. 6 *A*, *Bottom*). We analyzed individual autocorrelations to quantify and identify differences in the periodicity between PP1^+/−^ and PP1^−/−^ fish, but did not find strong periodicity in the z-positions from the correlograms in either genotype (*SI* *Appendix*, Fig. S7*A*). This raised the possibility that the fish’s z-position was not solely determined by light intensity. Next, we analyzed ΔZ values and found that upward and downward movements synchronized with the sine wave curves of short-wavelength light intensity were observed in PP1^+/−^ fish but not in PP1^−/−^ fish (Fig. 6*B*). Consistent with this, correlogram analyses also revealed periodicity following the 0.02 Hz sine waves in PP1^+/−^ but not in PP1^−/−^ fish (*SI* *Appendix*, Fig. S7*B*). Furthermore, the difference of the ΔZ data between PP1^+/−^ and PP1^−/−^ fish was analyzed by Fast Fourier Transform. Analysis revealed a peak at ~0.02 Hz in the mean trace of the power spectra for ΔZ of PP1^+/−^ fish, but not in PP1^−/−^ fish (Fig. 6*C*), with the difference quantitatively confirmed (Fig. 6*F*).

We successfully isolated “pure” opponent behaviors, upward and downward movements, driven solely by PP1-mediated photoreception, allowing us to evaluate whether the light-dependent behaviors involve the pineal–tegmentum pathway that conveys neural information derived from PP1. First, we recorded the movements of wild-type fish. Then, we extracted and visualized the neurons showing chromatic responses with light stimuli with a quick imaging procedure using a two-photon microscope. Based on the “map” of C-type tegmentum neurons, the neurons were selectively “ablated” by repeatedly scanning pixels with a two-photon laser (800 nm), and subsequent testing confirmed that the C-type responses were abolished (*SI* *Appendix*, Fig. S8 *A*–*D*). After the fish movements recovered from imaging and ablation procedures, behaviors were recorded again. A comparison of movements before and after laser ablation revealed that the ablation significantly reduced the behavioral periodicity between fish movements and changes in short-wavelength light intensities following sine wave curves (Fig. 6 *D* and *G* and *SI* *Appendix*, Fig. S8 *E* and *G*). In contrast, in fish that underwent sham ablation, no significant change was observed (Fig. 6 *E* and *H* and *SI* *Appendix*, Fig. S8 *F* and *H*). Therefore, ablation of C-type neurons in the tegmentum attenuated both upward and downward movements in response to gradual changes in wavelength composition. It should be noted that there were no significant differences in the magnitude of light-dependent locomotor activity before and after tegmental ablation at light intensities around both the peaks and troughs of the sine curve (*SI* *Appendix*, Fig. S9). These results indicate that neural information generated by PP1-based color opponency and conveyed via the tegmentum contributes to the decision-making underlying vertical movements.

### The Contribution of Pineal Photoreception to Vision-Based Light–Dark Preference.

Since we observed the integration of pineal and retinal light information in C-type tegmentum neurons (Fig. 3*L*), we further investigated the possibility that pineal photoreception influences light–dark preference decision-making through visual function (16). We investigated changes in fish population before and after the emergence of the shaded D-area, where an IR filter covers the area, preventing visible light from passing through (*SI* *Appendix*, Fig. S10*A*). Light with a spectral distribution appropriate for activating PP1 was used for the behavior test (*SI* *Appendix*, Fig. S10*B*). Depending on the light intensity, both intact WT and PP1^−/−^ fish exhibited different behaviors (*SI Appendix*, Fig. S10 *C*, *i*–*iii* and *E*, *i*–*iii*). Under weaker light conditions, the fish moved to the L-area after the “D-area” was darkened (*SI Appendix*, Fig. S10 *C*, *i* and *E*, *i*). In contrast, strong light conditions prompted the fish to move to the D-area (*SI Appendix*, Fig. S10 *C*, *iii* and *E*, *iii*). Under medium light intensity, WT fish showed no significant preference, whereas PP1^−/−^ fish demonstrated a significant preference for the L-area compared with WT fish (*SI Appendix*, Fig. S10 *C*, *ii* and *E*, *ii*). We also confirmed that the preference observed in intact fish was absent in enucleated fish (*SI Appendix*, Fig. S10 *D* *i*–*iii* and *F*, *i*–*iii*). These results suggest that nonvisual pineal photoreception influences “visual” function.

## Discussion

In this study, we established a light irradiation protocol to visualize PP1-specific responses in the pineal organ (Figs. 1 and 2). We found that responses generated by PP1-based color opponency are transmitted to tegmentum neurons, which are indirectly influenced by retinal photoreceptions (Fig. 3). Analyses of the projection patterns from the pineal to the tegmentum suggest that the pineal organ has a spatial resolution for light (Fig. 4). In addition, we found that the neural circuit from the pineal to the tegmentum, which processes light information captured by PP1, plays a role in regulating upward and downward movements in response to short-wavelength light changes under constant blue light (Figs. 5 and 6). This study provides physiological evidence that light information detected by a pineal opsin is transmitted to the brain and contributes to light-dependent behaviors in zebrafish larvae.

### Heterogeneity in Chromatic Ganglion Cells Showing the Response Property of PP1 Cells.

We found that some pineal ganglion cells showed chromatic responses derived from PP1-evoked photoresponses. Response-visualized images showed that PP1^−/−^ fish almost completely lost the cells showing Ca^2+^ decreases in response to UV light (Fig. 2 *B*, *vii*–*ix*). This result indicates that other pineal opsins do not significantly contribute to the calcium decrease in response to UV stimuli in our current experimental procedure. On the other hand, in PP1^+/+^ and PP1^+/−^ fish, several ganglion cells that exhibited clear calcium decreases in response to UV light stimuli did not exhibit calcium increases when exposed to visible light stimuli (Fig. 2 *B*, *i*–*vi*, cyan arrowheads). While the majority of calcium decreases in these ganglion cells in response to UV stimuli are likely caused by PP1, some cells did not fully exhibit the response properties of PP1 cells. One explanation for this observation could be the heterogeneity of inputs to the ganglion cells. Alternatively, if not all UV-induced calcium decreases observed in PP1^+/+^ and PP1^+/−^ fish are directly mediated by PP1, some of the deficits observed in PP1^−/−^ fish may reflect indirect effects of PP1 loss on pineal integrity or general physiology, which cannot be entirely excluded.

The zebrafish pineal organ exhibits visible-light sensitivity based on opsins, including exorhodopsin, long-wavelength-sensitive opsin, parapinopsin 2, and melanopsin (32–35). Each of these opsins is expressed exclusively with PP1 (34) and may cancel the PP1-evoked calcium increase by transmitting light information to ganglion cells that also receive light information from PP1 cells. In fact, we observed visible light-induced calcium decreases in pineal ganglion cells (Movie S1). In addition, in the pineal organ of PP1^−/−^ fish, we found several cells that exhibited visible light-induced small calcium increases but not UV-induced calcium decreases. These responses could be evoked by parietopsin, a visible light-sensitive opsin coexpressed with PP1 (9), as it is known to induce depolarization mediated by Go signaling (36, 37) (Fig. 2 *B*, *ix*, red arrowheads). Future studies will aim to investigate how other visible light-sensitive pineal opsins contribute to these observations. We also found that C-type responses in pineal ganglion cells were markedly reduced in PP1^−/−^ fish; however, our systematic pixel-based analyses still detected residual chromatic signals (Fig. 2*J*). These signals may reflect the residual calcium response profiles observed in PP1-expressing cells of PP1^−/−^ fish (*SI* *Appendix*, Fig. S2). The presence of these residual signals raises the possibility that a minor component of color opponency may be mediated by opsins other than PP1, although the molecular identity of the responsible opsin(s) remains an open question for future studies.

### “Chromatic” Neurons Exhibiting the Response Property of PP1 Cells Are Limited to the Tegmentum.

We found that PP1 photoresponses are transmitted to the tegmentum (Fig. 3). The pineal projections to the brain have been studied with histological techniques in several animals, including zebrafish (38–41). Previous reports using DiI and DiO, lipophilic carbocyanine tracers, indicated that projections from the pineal organ extend to multiple regions of the brain, including the thalamus, tectum, suprachiasmatic nucleus, and tegmentum in adult zebrafish (4). We did not observe neurons exhibiting strong “chromatic” properties in any region other than the tegmentum (Fig. 3 *G*, *i*–*vi*). Our findings in enucleated fish were consistent with this observation (Fig. 3*D*). Therefore, it seems unlikely that regions such as the tectum, thalamus, and suprachiasmatic nucleus, known to receive retinal light information, exhibit PP1-based chromatic properties due to strong retinal innervation. Consequently, we suggest that the tegmentum serves as the primary “center” for processing information from pineal PP1 photoreception, although this conclusion may be limited to the larval stages examined in this study. It remains an open question whether light information from pineal opsins other than PP1 is transmitted to multiple brain regions, and this will be explored in future studies.

### Possible Neural Pathway from the Retina to the Tegmentum and Its Significance in Spatial Resolution in Pineal Photoreception.

We investigated which eye contributes to the photoresponse in the C-type neurons of the left and right tegmentum by using fish with either eye enucleated. The results showed no strong bias in photoreception between the left and right eyes in the tegmentum neurons, whether contralateral or ipsilateral, during visible light stimuli (Fig. 4 *B* and *E*). However, while the calcium change profiles in response to UV stimuli were almost identical, those in response to visible light stimuli showed slight differences between contralateral and ipsilateral eye-enucleated fish. These observations suggest that the C-type tegmentum neurons receive weakly biased inputs from both ipsilateral and contralateral eyes. This indicates that the tegmentum neurons may be affected by neural responses in the arborization fields (AFs 1 to10), the direct projection area of retinal ganglion cells, including the tectum (AF10) and pretectum (AFs 1 to 9), with left and right spatial properties (17, 24, 42–44). In addition, from the viewpoint of not strongly biased innervation from the eye to the tegmentum, it may be reasonable to consider that most of the retinal inputs to tegmentum neurons are relayed via the entopeduncular nucleus (AF2 or 4)–habenula–interpeduncular nucleus pathway (45, 46), which is known to generate fear-related behaviors (47, 48).

It is generally understood that photoreception in pineal-related organs contributes to detecting light from the sky due to the dorsal location of pineal-related organs (36, 49, 50). The distribution of light in the sky is not uniform, especially around sunrise and sunset. The spatial resolution of pineal photoreception may serve as a “compass,” utilizing directional information obtained from the biased spatial distribution of sky light and/or the position of the sun. We previously suggested that PP1-based color opponency primarily functions during the daytime since it requires “strong” light to form photoequilibria between its two states: dark states and photoproducts (9). Through PP1-based color opponency, it may be possible to discriminate between regions with different spectral distributions, such as sunny areas exposed to direct sunlight and shady areas exposed to indirect sunlight (e.g., cloud-filtered and diffused sunlight). The spatial resolution in PP1-based color opponency could provide the ability to acquire spatial information about cloud distribution, thickness, and patterns in the sky, which could be useful for assessing weather conditions and moving to optimal locations for survival.

### Light-Dependent Up-and-Down Movements Likely Involve a Hierarchy in Fish Photosensory Systems.

We found that zebrafish exhibited short-wavelength light-dependent downward movements under a blue background, and these behaviors involve PP1-based photoreception (Fig. 5 *D* and *F*). This indicates that movements toward the bottom are partially driven by the conversion of UV-sensitive PP1 dark states to visible light-sensitive photoproducts upon UV light absorption. However, under dark background conditions, both PP1^+/−^ and PP1^−/−^ fish showed similar upward movements in response to UV light of sufficient intensity to facilitate the conversion from dark states of PP1 to photoproducts (Fig. 5 *E* and *G*). This upward movement is considered to reflect retina-based phototactic behavior during the local movement phase following light offset (31). Therefore, it is suggested that, under dark background conditions, zebrafish exhibited upward behavior possibly based on UV light reception in the retina, rather than downward behavior involving PP1 in the pineal organ in response to its UV light absorption. These observations suggest that zebrafish exhibit opposite behaviors to UV light under different background light conditions, which may result from a hierarchical interaction among multiple photosensory systems, including the pineal organ and eyes in zebrafish larvae, rather than being solely dependent on the photosensitivity and dynamic range of PP1 cells. In other words, the hierarchical interaction between pineal and retinal photoreceptions may contribute to the decision-making process regarding the direction of upward and downward movements. The residual color opponent responses observed in pineal ganglion cells of PP1^−/−^ fish (Fig. 2*J*) cannot be excluded in this context, although their functional contribution remains unclear.

We examined light-dependent behaviors and found that, under a blue background, long-wavelength light induced behaviors opposite to those triggered by short-wavelength light (*SI* *Appendix*, Fig. S6 *A*, *C*, and *E*). Specifically, no notable position change was observed at the initial onset of long-wavelength light, but its offset and subsequent onset induced downward and upward movements in both PP1^+/−^ and PP1^−/−^ fish. Although long-wavelength light is expected to revert the PP1 photoproducts to the dark state, the contribution of photoreception by PP1 photoproducts to these responses remains unclear, suggesting the possible involvement of other visible light-sensitive opsins. Note that light-dependent behaviors in darkness did not show opposing patterns in response to short- and long-wavelength light (Fig. 5*D* and *SI* *Appendix*, Figs. S5 and S6 *B* and *D*).

### Significance of the Temporal Window in Up-and-Down Movements for Detecting Gradual Changes in Wavelength Components.

We observed behavioral differences among genotypes in response to repeated light stimulation under background light (Fig. 5) and further characterized PP1-mediated behaviors via neural processing in the tegmentum using stimuli with gradual changes in light intensity (Fig. 6). This combination of light stimuli could be essential for advancing research on unknown photosensory systems in animals.

The downward movement in response to the first short-wavelength light onset was observed in both PP1^+/−^ and PP1^−/−^ fish (Figs. 5*E* and 6*A*). These observations suggest the involvement of unidentified opsins, other than PP1, in mediating behavioral responses to large or rapid changes in wavelength components and/or light intensity. In contrast, PP1-based color opponency is thought to contribute to the decision-making process for upward and downward behaviors in response to gradual changes in wavelength components. Periodic analyses between behavioral indices (such as z-position and ΔZ) and light intensity suggest that the essential role of PP1 photoreception is not to detect the absolute depth from the water surface, but to determine movement in response to light changes (*SI* *Appendix*, Fig. S7 *A* and *B*).

We also investigated the correlation coefficients between behavioral indices and light intensity and found that these correlations were never strong (*SI* *Appendix*, Fig. S11 *A* and *F*), suggesting that upward and downward movements lag behind the sine waves. To explore this further, we analyzed the time window for light change detection using trace smoothing. If behavior is evoked based on a temporal accumulation of photoreception by PP1, the correlation may be strengthened by smoothing ΔZ values over several time windows. The smoothed values of ΔZ represent cumulative behaviors, which reflect behaviors from the past to the present. Four types of time window-based smoothing analyses revealed that the negative correlations between the sine waves and the smoothed ΔZ values in WT and PP1^+/−^ fish were strongest at a 10-s time window (Fig. 6 *I*–*K* and *SI* *Appendix*, Fig. S11 *F*–*J*). In contrast, correlations between z-position and sine wave were not strongly affected by time window smoothing (*SI* *Appendix*, Fig. S11 *A*–*E*). These results suggest that upward and downward movements may depend on the processing of light information from PP1 cells over an approximately 10-s temporal window.

In this study, we provide insight into how light information derived from PP1-based color opponency contributes to decision-making for upward and downward movements through the integration of light information from both the pineal organ and the retina by tegmentum neurons. In particular, we demonstrated the presence of neural circuits connecting the pineal organ to tegmentum neurons by applying a light irradiation protocol that specifically evokes PP1-mediated responses. This approach, which combines the spectral properties of opsins with calcium imaging, may offer a method for investigating neural circuits.

## Materials and Methods

### Animals.

Zebrafish (*Danio rerio*) were obtained from the Zebrafish International Resource Center and the National BioResource Project Zebrafish. Zebrafish were maintained on a 14-h light/10-h dark cycle at 28.5 °C. Larvae were raised in E3 medium containing 5 mM NaCl, 0.17 mM KCl, 0.33 mM CaCl_2_, and 0.33 mM MgSO_4_. All animal procedures were approved by the Osaka Metropolitan University Animal Experiment Committee (approval no. S0032) and complied with Osaka Metropolitan University’s regulations on animal experiments.

### Generation of Transgenic Zebrafish.

To generate the pT2K-HuC-GCaMP6s-pAS plasmid, the GCaMP6s cDNA fragment was amplified by PCR from pGP-CMV-GCaMP6s (Addgene #40753) (51) and subcloned into the *HuC:Kaede* plasmid (18), which contains an SV40 polyadenylation signal (pAS). The resulting HuC-GCaMP6s-pAS fragment was then subcloned into the Tol2 vector pT2K-Dest-RfaF (52), which is derived from pT2KXIG (53), using the Gateway system (Thermo Fisher Scientific). Approximately 25 pg of plasmid DNA and 25 pg of Tol2 mRNA were coinjected into embryos at the one-cell stage.

Further details on experimental procedures and methods are provided in *SI Appendix*, *Methods*.

## Supplementary Material

Appendix 01 (PDF)

Movie S1. **Visualization of calcium dynamics in whole-brain imaging using image analysis.** Cells showing a calcium decrease (yellow) or increase (blue) in response to light stimulation targeting the pineal organ were visualized. However, most of the observed calcium responses are considered to be derived from retinal photoreception.

Movie S2. **Calcium dynamics in PP1 cells in response to a custom-designed light irradiation protocol based on the spectral properties of the two states of PP1, related to Figure 1.** PP1 cells exhibit a calcium decrease in response to 405-nm light. Under continuous 405-nm light, the same cells exhibit a calcium increase in response to 588-nm light.

## Data Availability

There are no data underlying this work.

## References

[1] G. M. Cahill, Circadian regulation of melatonin production in cultured zebrafish pineal and retina. Brain Res. **708**, 177–181 (1996). 8720875 10.1016/0006-8993(95)01365-2

[2] A. V. Gandhi, E. A. Mosser, G. Oikonomou, D. A. Prober, Melatonin is required for the circadian regulation of sleep. Neuron **85**, 1193–1199 (2015). 25754820 10.1016/j.neuron.2015.02.016 PMC4851458

[3] D. C. Klein, Arylalkylamine N-acetyltransferase: “The Timezyme”. J. Biol. Chem. **282**, 4233–4237 (2007). 17164235 10.1074/jbc.R600036200

[4] J. Yanez, J. Busch, R. Anadon, H. Meissl, Pineal projections in the zebrafish (Danio rerio): Overlap with retinal and cerebellar projections. Neuroscience **164**, 1712–1720 (2009). 19781601 10.1016/j.neuroscience.2009.09.043

[5] Y. Morita, Lead pattern of the pineal neuron of the rainbow trout (Salmo irideus) by illumination of the diencephalon. Pflugers Arch. **289**, 155–167 (1966). 5237284

[6] E. Dodt, E. Heerd, Mode of action of pineal nerve fibers in frogs. J. Neurophysiol. **25**, 405–429 (1962). 13886872 10.1152/jn.1962.25.3.405

[7] J. Falcon, H. Meissl, The photosensory function of the pineal organ of the pike (Esox-lucius L) correlation between structure and function. J. Comp. Physiol. **144**, 127–137 (1981).

[8] K. Uchida, Y. Morita, Spectral sensitivity and mechanism of interaction between inhibitory and excitatory responses of photosensory pineal neurons. Pflugers Arch. **427**, 373–377 (1994). 8072859 10.1007/BF00374547

[9] S. Wada , Color opponency with a single kind of bistable opsin in the zebrafish pineal organ. Proc. Natl. Acad. Sci. U.S.A. **115**, 11310–11315 (2018). 30322939 10.1073/pnas.1802592115 PMC6217433

[10] X. Chen , Brain-wide organization of neuronal activity and convergent sensorimotor transformations in larval zebrafish. Neuron **100**, 876–890 (2018). 30473013 10.1016/j.neuron.2018.09.042 PMC6543271

[11] F. Del Bene , Filtering of visual information in the tectum by an identified neural circuit. Science **330**, 669–673 (2010). 21030657 10.1126/science.1192949 PMC3243732

[12] E. Dreosti, N. Vendrell Llopis, M. Carl, E. Yaksi, S. W. Wilson, Left-right asymmetry is required for the habenulae to respond to both visual and olfactory stimuli. Curr. Biol. **24**, 440–445 (2014). 24508167 10.1016/j.cub.2014.01.016 PMC3969106

[13] Y. Kolsch , Molecular classification of zebrafish retinal ganglion cells links genes to cell types to behavior. Neuron **109**, 645–662 (2021). 33357413 10.1016/j.neuron.2020.12.003 PMC7897282

[14] A. Muto, M. Ohkura, G. Abe, J. Nakai, K. Kawakami, Real-time visualization of neuronal activity during perception. Curr. Biol. **23**, 307–311 (2013). 23375894 10.1016/j.cub.2012.12.040

[15] T. Roeser, H. Baier, Visuomotor behaviors in larval zebrafish after GFP-guided laser ablation of the optic tectum. J. Neurosci. **23**, 3726–3734 (2003). 12736343 10.1523/JNEUROSCI.23-09-03726.2003 PMC6742205

[16] B. B. Zhang, Y. Y. Yao, H. F. Zhang, K. Kawakami, J. L. Du, Left habenula mediates light-preference behavior in zebrafish via an asymmetrical visual pathway. Neuron **93**, 914–928 (2017). 28190643 10.1016/j.neuron.2017.01.011

[17] A. Kramer, Y. Wu, H. Baier, F. Kubo, Neuronal architecture of a visual center that processes optic flow. Neuron **103**, 118–132 (2019). 31147153 10.1016/j.neuron.2019.04.018

[18] T. Sato, M. Takahoko, H. Okamoto, HuC:Kaede, a useful tool to label neural morphologies in networks in vivo. Genesis **44**, 136–142 (2006). 16496337 10.1002/gene.20196

[19] E. Kawano-Yamashita , Activation of transducin by bistable pigment parapinopsin in the pineal organ of lower vertebrates. PLoS One **10**, e0141280 (2015). 26492337 10.1371/journal.pone.0141280 PMC4619617

[20] M. Koyanagi , Bistable UV pigment in the lamprey pineal. Proc. Natl. Acad. Sci. U.S.A. **101**, 6687–6691 (2004). 15096614 10.1073/pnas.0400819101 PMC404106

[21] A. Terakita , Counterion displacement in the molecular evolution of the rhodopsin family. Nat. Struct. Mol. Biol. **11**, 284–289 (2004). 14981504 10.1038/nsmb731

[22] E. Cau, A. Quillien, P. Blader, Notch resolves mixed neural identities in the zebrafish epiphysis. Development **135**, 2391–2401 (2008). 18550717 10.1242/dev.013482

[23] W. I. Davies , An extended family of novel vertebrate photopigments is widely expressed and displays a diversity of function. Genome Res. **25**, 1666–1679 (2015). 26450929 10.1101/gr.189886.115 PMC4617963

[24] E. Robles, E. Laurell, H. Baier, The retinal projectome reveals brain-area-specific visual representations generated by ganglion cell diversity. Curr. Biol. **24**, 2085–2096 (2014). 25155513 10.1016/j.cub.2014.07.080

[25] S. B. Udin, J. W. Fawcett, Formation of topographic maps. Annu. Rev. Neurosci. **11**, 289–327 (1988). 3284443 10.1146/annurev.ne.11.030188.001445

[26] B. Vigh , Nonvisual photoreceptors of the deep brain, pineal organs and retina. Histol. Histopathol. **17**, 555–590 (2002). 11962759 10.14670/HH-17.555

[27] M. Koyanagi, A. Terakita, Diversity of animal opsin-based pigments and their optogenetic potential. Biochim. Biophys. Acta **1837**, 710–716 (2014). 24041647 10.1016/j.bbabio.2013.09.003

[28] T. D. Lamb, Evolution of phototransduction, vertebrate photoreceptors and retina. Prog. Retin. Eye Res. **36**, 52–119 (2013). 23792002 10.1016/j.preteyeres.2013.06.001

[29] B. Shen , Functional identification of an opsin kinase underlying inactivation of the pineal bistable opsin parapinopsin in zebrafish. Zool. Lett. **7**, 1 (2021). 10.1186/s40851-021-00171-1 PMC7881645 33579376

[30] B. Shen , Light intensity-dependent arrestin switching for inactivation of a light-sensitive GPCR, bistable opsin. iScience **28**, 111706 (2025). 39925416 10.1016/j.isci.2024.111706 PMC11803233

[31] E. J. Horstick, Y. Bayleyen, J. L. Sinclair, H. A. Burgess, Search strategy is regulated by somatostatin signaling and deep brain photoreceptors in zebrafish. BMC Biol. **15**, 4 (2017). 28122559 10.1186/s12915-016-0346-2 PMC5267475

[32] J. Robinson, E. A. Schmitt, J. E. Dowling, Temporal and spatial patterns of opsin gene expression in zebrafish (Danio rerio). Vis. Neurosci. **12**, 895–906 (1995). 8924413 10.1017/s0952523800009457

[33] H. Mano, D. Kojima, Y. Fukada, Exo-rhodopsin: A novel rhodopsin expressed in the zebrafish pineal gland. Brain Res. Mol. Brain Res. **73**, 110–118 (1999). 10581404 10.1016/s0169-328x(99)00242-9

[34] M. Koyanagi , Diversification of non-visual photopigment parapinopsin in spectral sensitivity for diverse pineal functions. BMC Biol. **13**, 73 (2015). 26370232 10.1186/s12915-015-0174-9 PMC4570685

[35] D. Sapede, C. Chaigne, P. Blader, E. Cau, Functional heterogeneity in the pineal projection neurons of zebrafish. Mol. Cell. Neurosci. **103**, 103468 (2020). 32027966 10.1016/j.mcn.2020.103468

[36] C. Y. Su , Parietal-eye phototransduction components and their potential evolutionary implications. Science **311**, 1617–1621 (2006). 16543463 10.1126/science.1123802

[37] S. Wada , Insights into the evolutionary origin of the pineal color discrimination mechanism from the river lamprey. BMC Biol. **19**, 188 (2021). 34526036 10.1186/s12915-021-01121-1 PMC8444496

[38] J. Yanez, R. Anadon, Afferent and efferent connections of the habenula in the rainbow trout (Oncorhynchus mykiss): An indocarbocyanine dye (DiI) study. J. Comp. Neurol. **372**, 529–543 (1996). 8876451 10.1002/(SICI)1096-9861(19960902)372:4<529::AID-CNE3>3.0.CO;2-6

[39] J. Yanez, R. Anadon, Neural connections of the pineal organ in the primitive bony fish Acipenser baeri: A carbocyanine dye tract-tracing study. J. Comp. Neurol. **398**, 151–161 (1998). 9700564 10.1002/(sici)1096-9861(19980824)398:2<151::aid-cne1>3.3.co;2-9

[40] J. Yanez, R. Anadon, B. I. Holmqvist, P. Ekstrom, Neural projections of the pineal organ in the larval sea lamprey (Petromyzon marinus L.) revealed by indocarbocyanine dye tracing. Neurosci. Lett. **164**, 213–216 (1993). 8152603 10.1016/0304-3940(93)90894-q

[41] J. Yanez, T. Suarez, A. Quelle, M. Folgueira, R. Anadon, Neural connections of the pretectum in zebrafish (Danio rerio). J. Comp. Neurol. **526**, 1017–1040 (2018). 29292495 10.1002/cne.24388

[42] F. Kubo , Functional architecture of an optic flow-responsive area that drives horizontal eye movements in zebrafish. Neuron **81**, 1344–1359 (2014). 24656253 10.1016/j.neuron.2014.02.043

[43] M. Kunst , A cellular-resolution atlas of the larval zebrafish brain. Neuron **103**, 21–385 (2019). 31147152 10.1016/j.neuron.2019.04.034

[44] Y. Wu, M. Dal Maschio, F. Kubo, H. Baier, An optical illusion pinpoints an essential circuit node for global motion processing. Neuron **108**, 722–734 (2020). 32966764 10.1016/j.neuron.2020.08.027

[45] H. Okamoto, M. Agetsuma, H. Aizawa, Genetic dissection of the zebrafish habenula, a possible switching board for selection of behavioral strategy to cope with fear and anxiety. Dev. Neurobiol. **72**, 386–394 (2012). 21567982 10.1002/dneu.20913

[46] R. K. Cheng, S. Krishnan, Q. Lin, C. Kibat, S. Jesuthasan, Characterization of a thalamic nucleus mediating habenula responses to changes in ambient illumination. BMC Biol. **15**, 104 (2017). 29100543 10.1186/s12915-017-0431-1 PMC5670518

[47] M. Agetsuma , The habenula is crucial for experience-dependent modification of fear responses in zebrafish. Nat. Neurosci. **13**, 1354–1356 (2010). 20935642 10.1038/nn.2654

[48] E. R. Duboue, E. Hong, K. C. Eldred, M. E. Halpern, Left habenular activity attenuates fear responses in larval zebrafish. Curr. Biol. **27**, 2154–2162.e2153 (2017). 28712566 10.1016/j.cub.2017.06.017 PMC5570455

[49] A. Oksche, Survey of the development and comparative morphology of the pineal organ. Prog. Brain Res. **10**, 3–29 (1965). 14281614 10.1016/s0079-6123(08)63445-7

[50] E. Solessio, G. A. Engbretson, Antagonistic chromatic mechanisms in photoreceptors of the parietal eye of lizards. Nature **364**, 442–445 (1993). 8332214 10.1038/364442a0

[51] T. W. Chen , Ultrasensitive fluorescent proteins for imaging neuronal activity. Nature **499**, 295–300 (2013). 23868258 10.1038/nature12354 PMC3777791

[52] H. Nojima , Syntabulin, a motor protein linker, controls dorsal determination. Development **137**, 923–933 (2010). 20150281 10.1242/dev.046425

[53] K. Kawakami , A transposon-mediated gene trap approach identifies developmentally regulated genes in zebrafish. Dev. Cell **7**, 133–144 (2004). 15239961 10.1016/j.devcel.2004.06.005

