# Supplementary material for: Neural circuits for decision-making based on pineal photoreception in zebrafish
Source: Proc Natl Acad Sci U S A. 2026 Mar 31;123(14):e2520290123. doi: 10.1073/pnas.2520290123 (PMC13056136; doi:10.1073/pnas.2520290123)
Supplement: Supplementary file 1 — Appendix 01 (PDF) [file pnas.2520290123.sapp.pdf]

Supporting Information for
Neural circuits for decision-making based on pineal photoreception in zebrafish

Seiji Wada[1,2,4], Yuki Yamamoto[1], Tomoka Saito[1], Masahiko Hibi[3], Mitsumasa Koyanagi[1,2], and Akihisa Terakita[1,2,*]

[1]Department of Biology, Graduate school of Science, Osaka Metropolitan University, Osaka 558-8585, Japan
[2]The OMU Advanced Research Institute for Natural Science and Technology, Osaka Metropolitan University, Osaka 558-8585, Japan
[3]Department of Biological Science, Graduate School of Science, Nagoya University, Furo, Chikusa, Nagoya, Aichi 464-8602, Japan.
[4]Present address: Department of Chemistry and Biological Science, College of Science and Engineering, Aoyama Gakuin University, 5-10-1 Fuchinobe, Chuo-ku, Sagamihara-shi, Kanagawa 252-5258, Japan

*Corresponding author: Akihisa Terakita
Email: terakita@omu.ac.jp

This PDF file includes:

Methods
Legends for Movies S1 to S2
Figures S1 to S11

Methods

Two-photon imaging

Zebrafish larvae (5–6 days post-fertilization (dpf), or 10–11 dpf for quick imaging used for laser ablation to minimize hypoxia-induced mortality) were anesthetized in E3 medium containing 0.002% Tricaine (MS222; Sigma) and embedded dorsal side up in 1.5% low-melting-point agarose gel prepared in Ringer's solution (116 mM NaCl, 2.9 mM KCl, 1.8mM $CaCl_2$, and 5mM HEPES, pH 7.2) on 35-mm glass-bottom dishes (Iwaki). Ringer's solution was added to prevent drying.

Two-photon calcium imaging was performed on transgenic fish, *Tg(parapinopsina(pp1):GCaMP6s)^{zf3423}* (1) and *Tg(elavl3(HuC):GCaMP6s)^{nub126},* using a multiphoton laser scanning microscope (FVMPE-RS; Olympus). GCaMP6s was excited using a Mai Tai HP DeepSee IR laser (Spectra-Physics), and both wavelength and laser intensity were controlled with FLUOVIEW software (Olympus). XYZT imaging settings were as follows: the ROI for imaging was 512 (X) × 256 (Y) pixels (1 pixel = ~0.5 × 0.5 μm^2) and 20 planes (Z, 4.5 μm step) in imaging for the pineal organ (Figures 1, 2, and S2). 512 (X) × 150 (Y) pixels (1 pixel = ~1 μm^2) and 30 planes (Z, 2.2 μm step) for the tegmentum (Figures 3 and 4). 512 (X) × 512 (Y) pixels (1 pixel = ~1 μm^2), and 20 planes (Z, 4.5 μm step) for the whole brain (Figures 3 and 4). Whole-brain imaging was conducted in three separate layers and then merged (total depth: 270 μm). All XYZT scans were acquired from the ventral to the dorsal side. For whole-brain imaging, three separate layers were imaged independently. Acquisition of a single volumetric frame consisting of 20 planes required approximately 2 s, resulting in temporal offsets even within a single frame. Therefore, because of these temporal offsets, we avoided comparisons and discussions of response kinetics based on frame-to-frame (two-point) changes in calcium levels across different brain regions. To visualize the C-type tegmentum neurons for laser ablation, quick imaging was performed in larvae following the behavioral assay. The imaging ROI was 512 (X) × 200 (Y) pixels (1 pixel = ~0.5 × 0.5 μm^2) and 10 planes (Z, 4.5 μm step).

The ROIs for stimulation with 405-nm (0.2 mW, OBIS; Coherent) and 588-nm (2 mW, Sapphire; Coherent) lasers were 160 (X) × 160 (Y) pixels (1 pixel = ~0.5 × 0.5 μm^2) in the pineal imaging (Figures 1, 2) and quick imaging (Figure S8), and 80 (X) × 80 (Y) pixels (1 pixel = ~1 μm^2) in the whole-brain and tegmentum imaging (Figures 3, 4). For separate stimulation of the left and right side of the pineal organ, ROIs were set at 40 (X) × 80 (Y) pixels (1 pixel = ~1 μm^2) positioned on each side (Figure 4C, F, and G; Figure S4).

Image analysis

All imaging data were acquired using FLUOVIEW software (Olympus) and analyzed by Image J/Fiji. Original 16-bit hyperstack images were converted to 8-bit hyperstack images. Images were averaged based on light conditions: 50 frames for pineal imaging, and 25 frames for whole-brain and tegmentum imaging. For imaging using *Tg(elavl3(HuC):GCaMP6s)* in the pineal organ, whole brain, or tegmentum, these averaged images were used for further processing. Specifically, based on the 50- or 25-frame averaged raw images, three ΔF/F images representing calcium decreases in response to UV ($\Delta F/F_{1 \text{ to } 3}$) and three ΔF/F images representing calcium increases in response to visible light ($\Delta F/F_{4 \text{ to } 6}$) were generated. Each set of images was averaged, and the standard error image was subtracted to create the final $\Delta F/F_{405}$ and $\Delta F/F_{588}$ images. To identify C-type neurons, a logical AND operation was performed between the $\Delta F/F_{405}$ and $\Delta F/F_{588}$ images, resulting in "C-type" images. C-type neurons were defined as pixels in which the trial-averaged ΔF/F was smaller than −SEM for UV responses and larger than +SEM for visible-light responses (SEM = SD/√3 across three trials). These images were processed with a median filter and binarized to define ROIs used for calcium dynamics extraction in C-type neurons. The visualization of C-type neurons in images obtained from quick imaging was performed by merging the images representing calcium decreases in response to UV and increases in response to visible light (Figure S8).

Surgical treatment, laser ablation, and sham operation

Prior to enucleation, larvae were embedded in 1.5% low-melting-point agarose in Ringer's solution containing 0.004% Tricaine (MS222; Sigma). Once the agarose solidified, the eye was removed using an insect pin, and the larva was transferred to Ringer's solution. Imaging was conducted after spontaneous swimming behavior had resumed (approximately 0.5–2 h later). Pineal ablation with an IR laser was performed prior to imaging in Figure 3E. The IR laser (800 nm, 10% intensity) was scanned five times for 10 s each over a 50 (X) × 1 (Y) pixel region on the posterior side of the pineal organ, where axons of pineal ganglion cells are localized.

For laser ablation of C-type neurons in the tegmentum, target neurons were first identified via quick imaging. Pixel scanning with the IR laser (800 nm, 10%, 1 s) was then performed three times at the center of the visualized neurons. To confirm successful ablation, quick imaging was repeated. If C-type neurons remained visible, the procedure was repeated (Figure S8A). Once the neurons were no longer detectable, the larva was transferred to Ringer's

solution for recovery. In sham operations, the same procedure followed, except that pixel scanning was randomly applied to three unrelated locations after quick imaging.

Three-dimensional behavior recording and analysis

Light-dependent behaviors of zebrafish were assessed in a transparent arena ($5 \times 5 \times 5$ cm^3). Behavioral recordings were performed using a CCD camera (Basler, acA1600-20um) equipped with a bi-telecentric lens (Moritex, MTL-18011C-006). The camera was modified by attaching an IR-pass filter (Fujifilm, IR-76) that blocks visible light while allowing IR light to pass. Recordings were conducted under IR illumination provided by custom-made IR light sources positioned above and to the side of the arena. A side view of the arena was captured through reflection via a hot mirror (Edmund optics, #43-958), allowing simultaneous acquisition of both side and bottom views in a single camera feed.

From 5 dpf, larvae were fed with paramecia. Before behavioral recordings, 10–11 dpf larvae were placed in the arena and initially maintained under standard white LED lighting until they swam near the surface. Recordings began immediately after the white LED light was turned off. After 30 s of continuous darkness, the test or background light was turned on. These lights were delivered using a multi-LED light system (CoolLED, pe-4000). Repeated light exposures and sine wave intensity changes were programmed using the default settings in the system. Fish trajectories were analyzed using EthoVision software (Noldus).

Light-dark preference test

Behavioral recordings were performed using 7 dpf larvae, following previous studies that employed this developmental stage for visual behavioral assays (2). The larvae were fed with paramecia for 5 to 7 dpf. An agarose gel molded in a 35-mm dish was used as the behavioral arena. An IR light source was placed beneath the arena, and behavior was recorded using a CCD camera (Basler, acA1600-20um). The 7 dpf larvae were introduced into the arena and their behavior was recorded under each light condition for 5 min. After this period, half of the upper surface of the arena was covered with an IR filter (Dark area). Videos were analyzed using UMATracker (3) to obtain the coordinates of all individuals, and the number of larvae in the illuminated and dark areas was counted. All experiments were conducted with 10 individuals.

Quantification and Statistical Analysis

The area of cells visualized in ΔF/F405, ΔF/F588, and C-type images was quantified using ImageJ/Fiji software. Statistical analyses for these data (Figure 2E, F, and I; Figure 3F) were conducted using Dunnett's tests in Python 3. Sample sizes (n, the number of individuals) are indicated in the graphs, along with the mean and standard error of the mean (SEM). The statistical evaluation of calcium change profiles was performed using paired *t*-tests in Microsoft Excel and visualized as heatmaps generated with ImageJ/Fiji software (Figure 3H, I, and L; Figure 4D–G). Behavioral indices between $PP1^{+/-}$ and $PP1^{-/-}$ (Figure 5D–G; Figure 6A–C, F), as well as those before and after laser ablation or sham treatment (Figure 6D, E, G, and H), were calculated using Microsoft Excel following trajectory analysis by EthoVision software. Autocorrelation and FFT analyses were performed using Python 3. Correlation coefficients were calculated using Microsoft Excel. Statistical tests for behavioral indices were conducted using Wilcoxon rank sum tests in IGOR Pro software (Figure 5F and G; Figure 6F and I) or paired *t*-tests in Microsoft Excel (Figure 6G, H, J, and K; Figure S9). The ratio of larvae occupying the D area in the light preference tests was calculated using Microsoft Excel following trajectory analysis by UMATracker, and paired *t*-tests were conducted in Microsoft Excel (Figure S10C–F). For the time-resolved calcium profiles (Figure 3H, I, and L; Figure 4D–G), paired *t*-tests were performed at each time point to visualize transient differences during and after stimulation. Because these analyses were intended solely for descriptive visualization rather than statistical inference, *p*-values were not corrected for multiple comparisons and were interpreted qualitatively.

Legends for Movies S1 to S2

Movie S1. Visualization of calcium dynamics in whole-brain imaging using image analysis.

Cells showing a calcium decrease (yellow) or increase (blue) in response to light stimulation targeting the pineal organ were visualized. However, most of the observed calcium responses are considered to be derived from retinal photoreception.

Movie S2. Calcium dynamics in PP1 cells in response to a custom-designed light irradiation protocol based on the spectral properties of the two states of PP1, related to Figure 1.

PP1 cells exhibit a calcium decrease in response to 405-nm light. Under continuous 405-nm light, the same cells exhibit a calcium increase in response to 588-nm light.

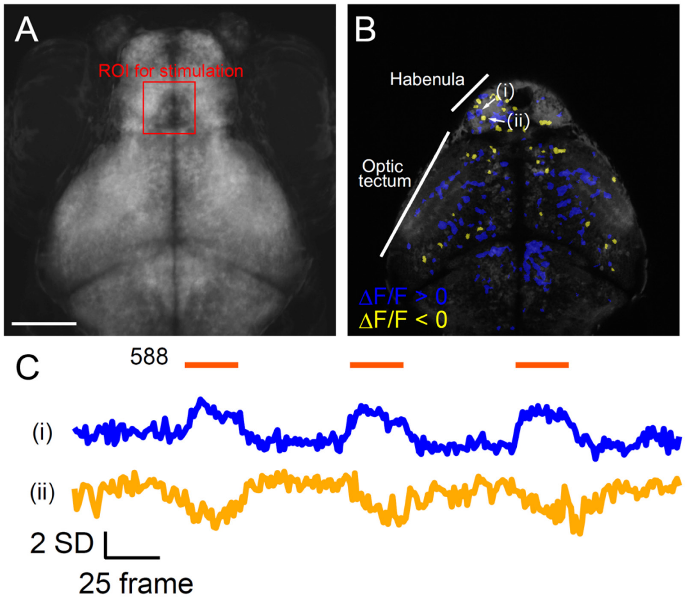

Figure S1. Difficulties in the investigation of pineal-innervated neurons in the larval zebrafish brain.

(A) Fluorescence image of *Tg(elavl3(HuC):GCaMP6s)*. A region of interest (ROI) for stimulation was set over the pineal organ. (B) Cells visualized by image analysis showing calcium decrease (yellow) and increase (blue). Laser scanning of the pineal organ induced reproducible calcium changes in brain regions innervated by the retina. (C) Representative profiles showing reproducible calcium changes in habenula neurons ((i) and (ii) in (B)). Scale bar: 100 µm.

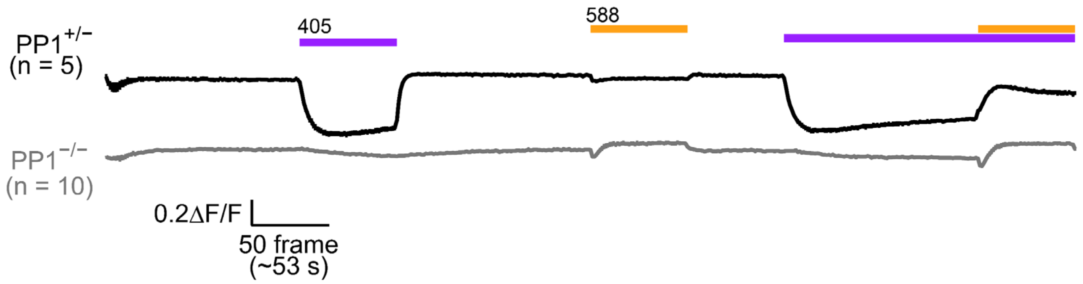

Figure S2. Analysis of visible-light responses based on the molecular properties of PP1 under different light conditions.

PP1-expressing cells of *Tg(pp1:GCaMP6s)* larvae exhibited a significant decrease in intracellular calcium levels in response to UV stimulation (405 nm; purple bars) in $PP1^{+/-}$ fish (n = 5), but not in $PP1^{-/-}$ fish (n = 10), indicating PP1 photoconversion from the dark state to the active photoproduct upon UV exposure causes a reduction in intracellular calcium level . A visible-light exposure (588 nm; yellow bars) under UV-free conditions induced a slight calcium decrease in $PP1^{+/-}$ fish, whereas no detectable response was observed in $PP1^{-/-}$ fish. In contrast, under UV background illumination, visible-light stimulation elicited a calcium increase in $PP1^{+/-}$ fish, indicating that a shift in the PP1 photoequilibrium toward the state containing a smaller proportion of photoproduct causes an increase in intracellular calcium level. It should be noted that the mechanism underlying the visible light-induced calcium decrease mediated by PP1 photoreception, in the absence of UV, remains unclear. One possible explanation is that photosensitivity to the two-photo excitation laser (i.e. blue background light) transiently increases due to rapid increase in the PP1 dark state, which is caused through visible light-induced back-reaction of the shutoff-element-mediated (e.g. arrestin-mediated) “inactivated photoproduct". Fluorescence values are normalized to the average of frames 51-100 (50 frames before the initial UV exposure). Experiments were performed using 5-6 dpf zebrafish larvae. Error bars show the SEM. XYZT imaging, rather than continuous single-plane two-photon scanning used in our previous study (1), was employed in this study.

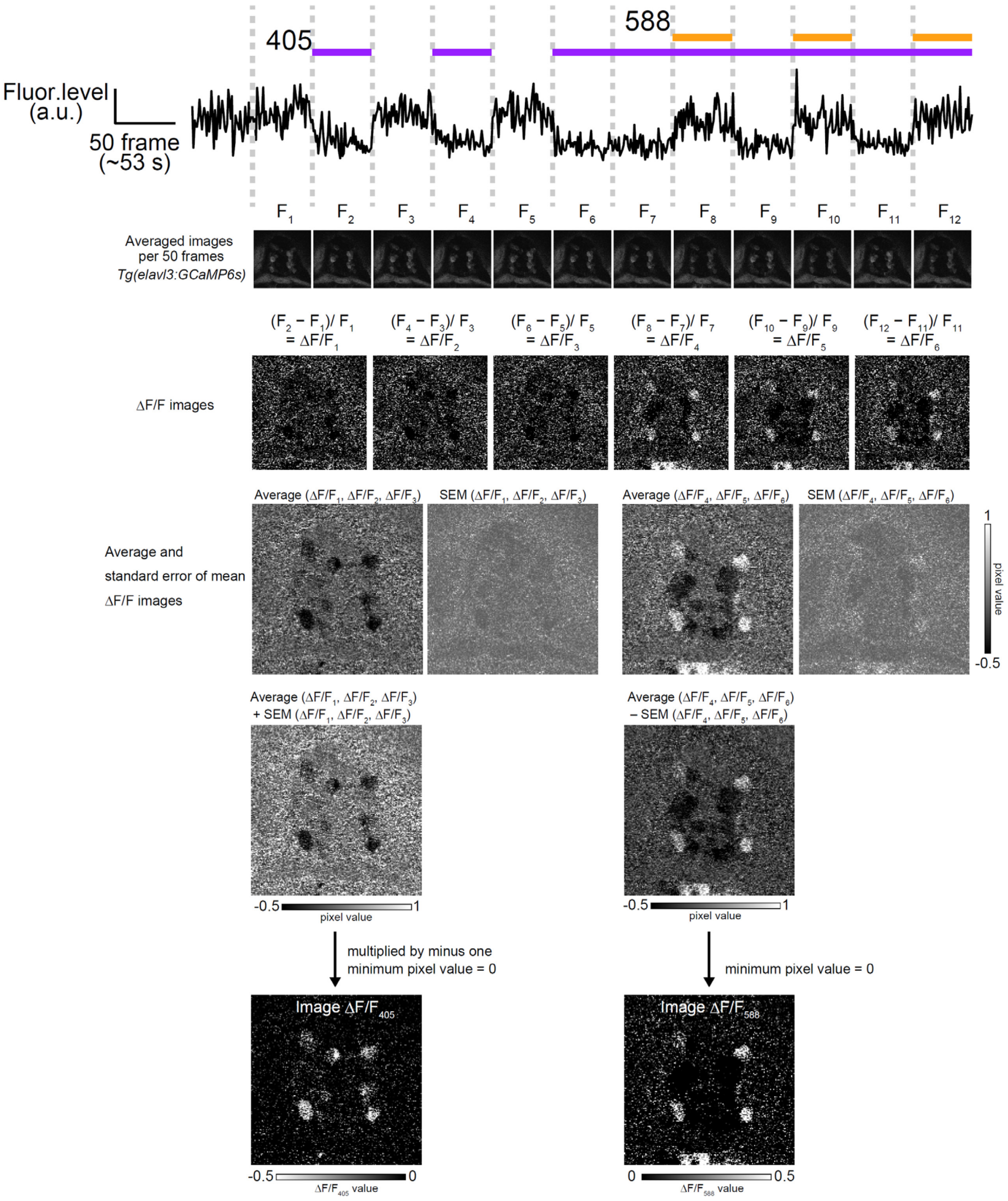

Figure S3. Detailed workflow of image analysis, related to Figures 2 and 3.

Single-plane time-series images of pineal ganglion cells from a *Tg(elavl3:GCaMP6s)* larva were used. Averaged images (dorsal view) were generated every 50 frames (25 frames in the case of whole-brain or tegmentum imaging), from which three ΔF/F images (32-bit floating-point images) each were produced for UV and visible-light stimulation. These images were then averaged to generate a mean image and the corresponding standard error of the mean (SEM) image. To focus on negative ΔF/F responses to UV light, the SEM image was added to reduce large noise components arising from spontaneous neuronal activity. In contrast, to emphasize positive ΔF/F responses to visible light, the SEM image was subtracted. Final images were displayed using a dynamic range optimized for the calcium-level changes of interest. After conversion to 8-bit images, a logical AND operation was applied to generate images representing C-type pixels (see Figure 2G, H).

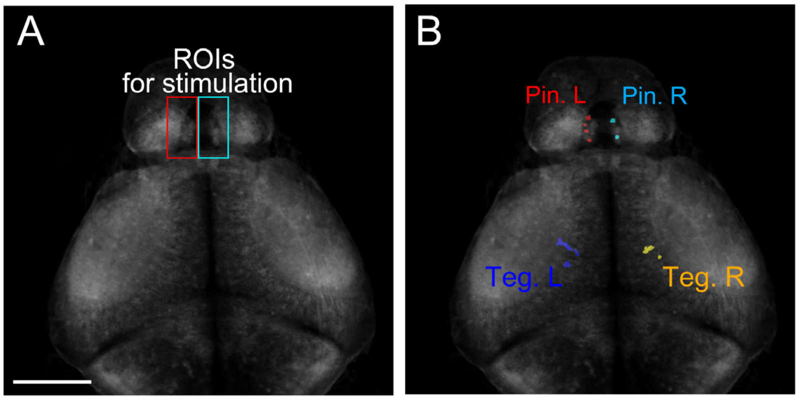

Figure S4. Representative images showing C-type neurons with separate color visualization, related to Figure 4.

(A) ROIs for stimulation used in Figure 4C, F, and G were separately set over the left and right sides of the pineal organ. (B) ROIs used for obtaining fluorescent values based on calcium level in C-type neurons analyses. Scale bar: 100 μm.

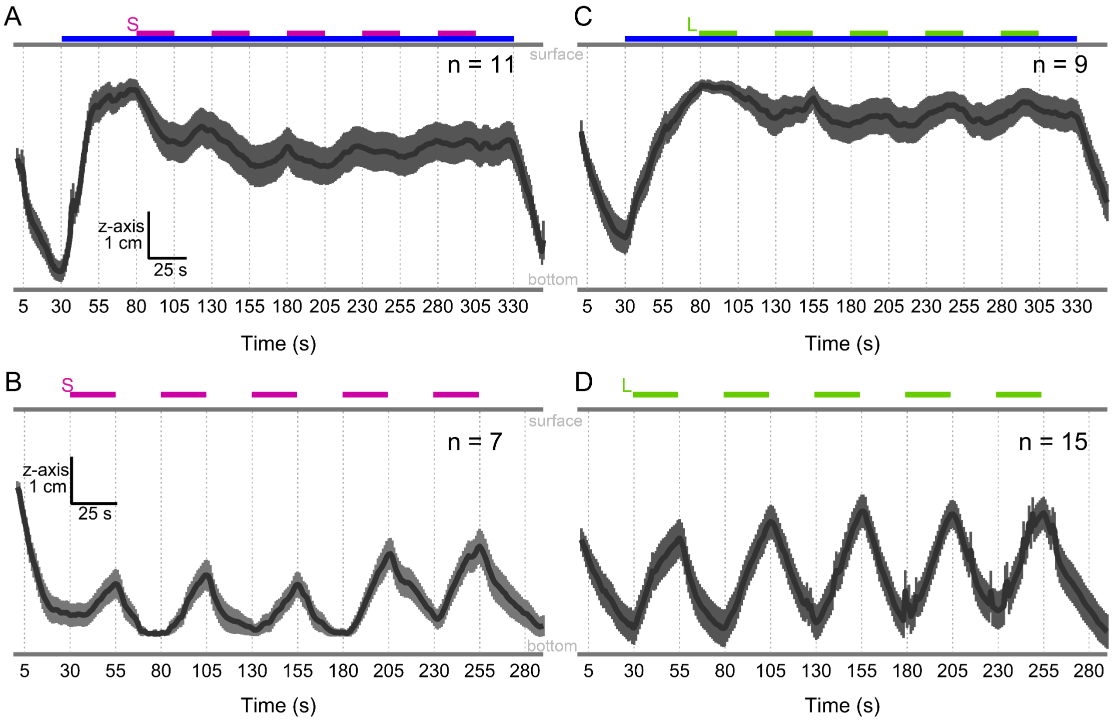

Figure S5. Differences in wild-type larval behavior under various light conditions.

Exposure to short-wavelength light (see Figure 5A) elicited downward movements in larvae at 10–11 dpf under conditions with blue background light (A, n = 11), whereas upward movements were observed under conditions without blue background light (B, n = 7; dark conditions). On the other hand, exposure to long-wavelength light (see Figure S6A) elicited upward movements regardless of the presence (C, n = 9) or absence (D, n = 15) of blue background light. These results indicate that larval behavioral responses to short-wavelength light are modulated by the presence or absence of blue background light. Error bars show the SEM.

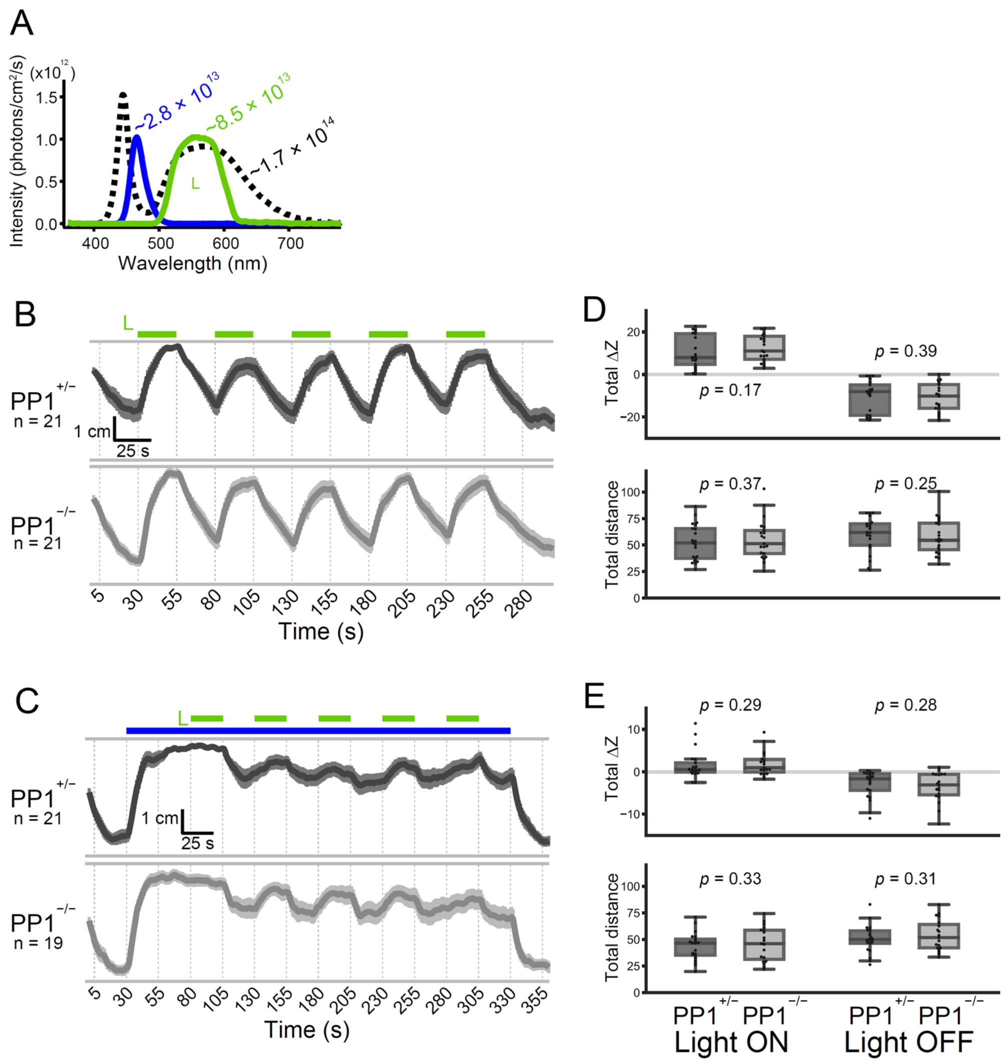

Figure S6. Long-wavelength light-dependent behaviors in PP1-deficient fish and siblings, related to Figure 5.

(A) Spectral distributions of light used in behavior tests. Long-wavelength light (L, green) was used as the stimulus. A standard white LED (dotted curve) was employed to maintain fish near the surface before recordings. Blue light was used for the background light. (B, C) Behavioral traces showing fish z-position changes in response to the L light exposure under dark (B) and blue (C) background conditions. Error bars show the SEM. (D, E) Quantification and comparison of total ΔZ (top) and distance (bottom) in (D) and (E) during the L light onset or offset between $PP1^{+/-}$ and $PP1^{-/-}$ fish (Wilcoxon rank sum test).

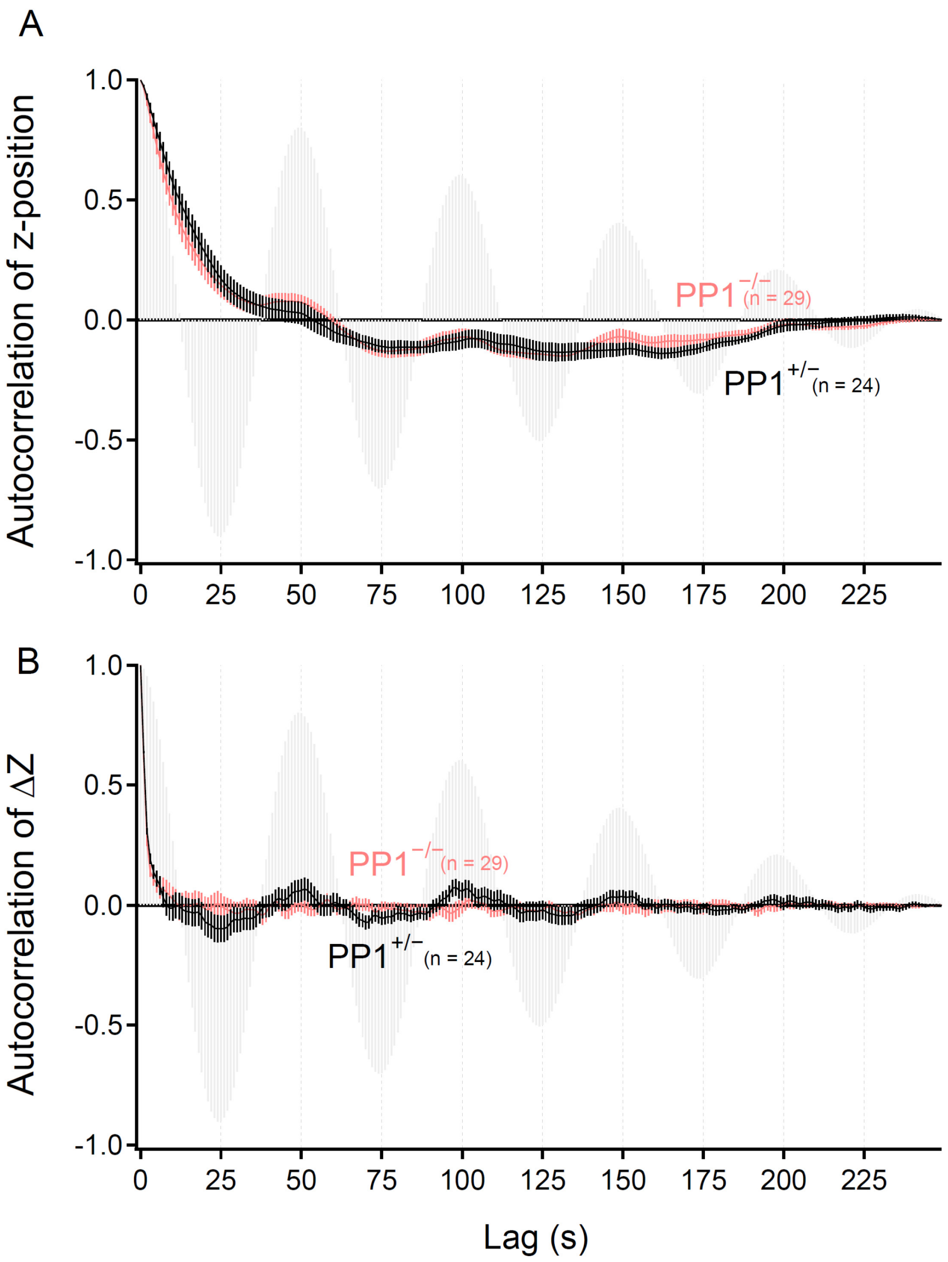

Figure S7. Autocorrelation analyses of behavioral indices corresponding to short-wavelength component changes following 0.02 Hz sine waves, related to Figure 6.

(A, B) Correlograms of z-position (A) and ΔZ (B) comparing $PP1^{+/-}$ (black) and $PP1^{-/-}$ (red) fish. The correlogram of ΔZ in $PP1^{+/-}$ fish (B, black) exhibits positive and negative correlations every 25 s, consistent with the light changes following the 0.02 Hz sine wave. Error bars show the SEM.

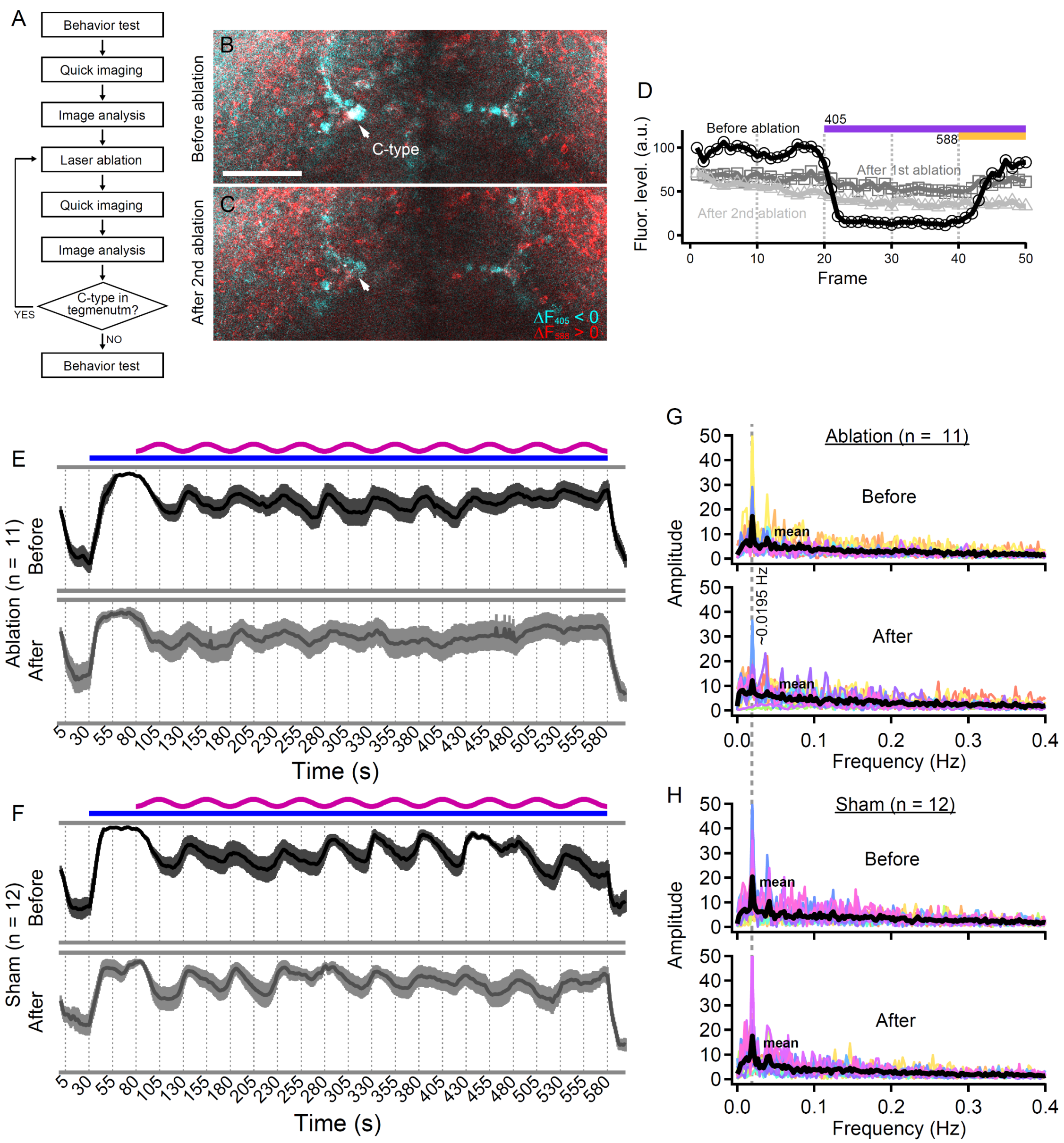

Figure S8. Laser ablation followed by behavior tests, related to Figure 6.

(A) Flow of the experiments. (B) Merged images before (top) and after (bottom) laser ablation of C-type tegmentum neurons. Decreases and increases in response to UV (cyan) and visible light (red) are visualized based on ΔF/F pixel values. Scale bar: 50 µm. (C) Calcium profiles before and after laser ablation in the same ROI (arrows in B). (E, F) Comparison of behavioral traces showing changes in fish z-position in response to sine wave-modulated short-wavelength light changes under a blue background before and after laser ablation of C-type tegmentum neurons (E) or sham treatment (F). Error bars show the SEM. (G, H) Power spectra representing periodicity in ΔZ from FFT analyses before and after laser ablation (G) or sham treatment (H).

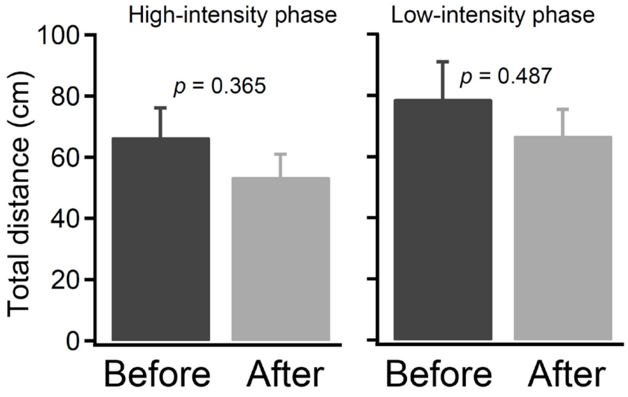

Figure S9. Locomotor activity in response to sine wave-modulated short-wavelength light.

Comparison of the total distance traveled before and after laser ablation of C-type tegmentum neurons during the high- and low-intensity phases (peaks and troughs) of a sine-wave-short-wavelength light exposure under blue background light (n = 11, paired *t*-test). Error bars show the SEM.

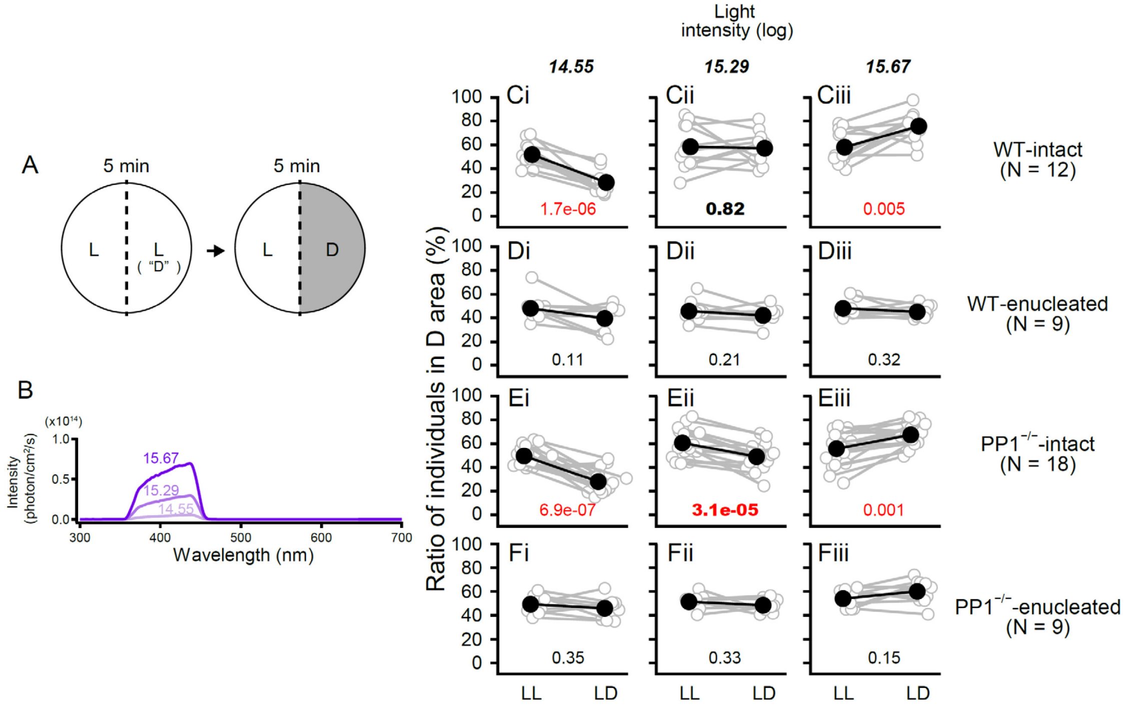

Figure S10. Light preference test using WT and PP1$^{-/-}$ fish.

(A) Schematic overview of the experiments for testing light preference. During the first 5 min, fish were exposed to each light condition in B. Next, the "D" area was darkened by covering it with an IR filter. The number of individuals in the dark area was counted. (B) Spectral distribution of the light conditions used in the tests. (C) Light intensity-dependent preference changes. WT and PP1$^{-/-}$ fish preferred the L- and D-area in the weaker and stronger light conditions (Ci-Ciii). The intensity-dependent preference was also dependent on the presence or absence of eyes (Di-Diii, Fi-Fiii). A clear preference was observed in PP1$^{-/-}$ but not in WT fish, under medium light conditions (paired *t*-test). All experiments were conducted with 10 individuals. The number "N" indicates the number of independent experiments.

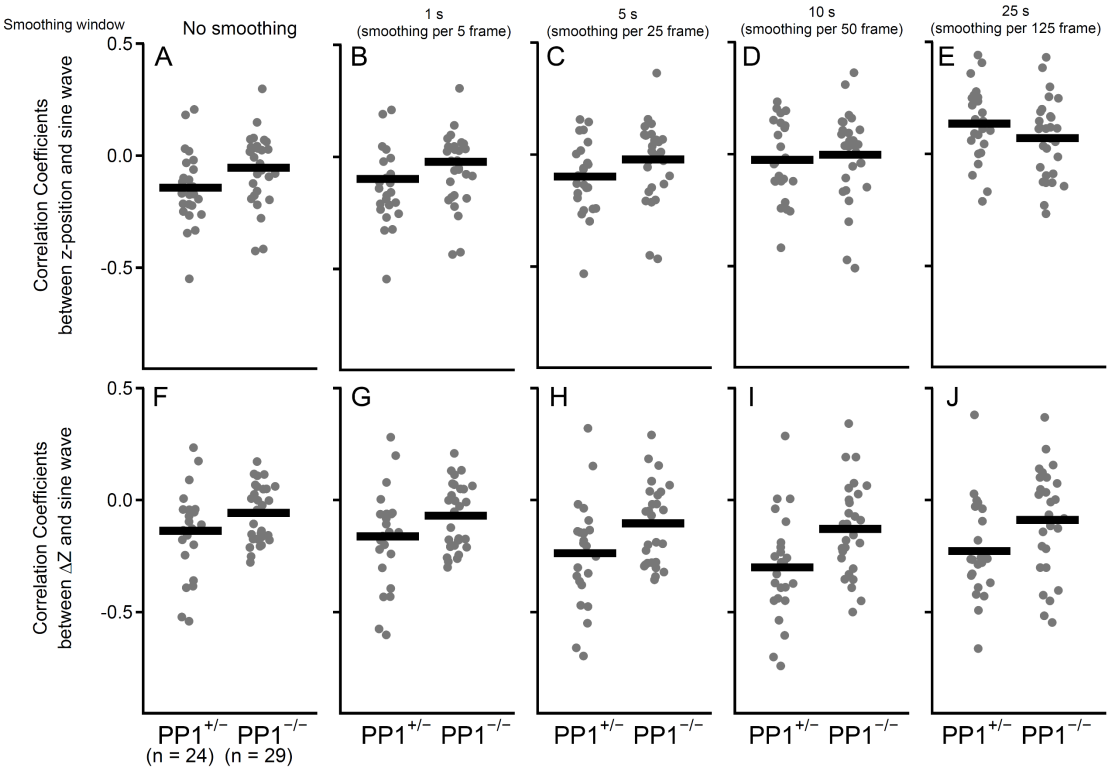

Figure S11. Correlation coefficients between behavioral indices and light intensity across several time windows based on behavioral trace smoothing, related to Figure 6.

(A-E) Correlation coefficients between raw (A) or smoothed (B-E) z-position of fish and light intensity in both genotypes. (F-J) Correlation coefficients between raw (F) or smoothed (G-J) ΔZ and z-position of fish and light intensity. Bold horizontal bars represent the mean.

SI references

1. Wada S*, et al.* (2018) Color opponency with a single kind of bistable opsin in the zebrafish pineal organ. *Proc Natl Acad Sci U S A* 115(44):11310-11315.
2. Zhang BB, Yao YY, Zhang HF, Kawakami K, & Du JL (2017) Left Habenula Mediates Light-Preference Behavior in Zebrafish via an Asymmetrical Visual Pathway. *Neuron* 93(4):914-928 e914.
3. Yamanaka O & Takeuchi R (2018) UMATracker: an intuitive image-based tracking platform. *J Exp Biol* 221(Pt 16).
